# Checklist of the subfamily Adoncholaiminae Gerlach and Riemann, 1974 (Nematoda: Oncholaimida: Oncholaimidae) of the world: genera, species, distribution, and reference list for taxonomists and ecologists

**DOI:** 10.3897/BDJ.4.e6577

**Published:** 2016-01-20

**Authors:** Daisuke Shimada

**Affiliations:** ‡ Department of Biology, Keio University, 4-1-1, Hiyoshi, Yokohama, 223-8521, Japan

**Keywords:** aquatic nematodes, marine, brackish, freshwater, oncholaimid, synonymy, taxonomy, systematics, nomenclature, ecology

## Abstract

**Background:**

Adoncholaiminae is one of the seven subfamilies in the free-living aquatic nematode family Oncholaimidae. Nematodes in Adoncholaiminae are found from various water environment of the world. However, a checklist of all Adoncholaiminae species including full literature, especially information of experimental (not taxonomic) works, has not been updated for more than 40 years.

**New information:**

A revised checklist of the subfamily Adoncholaiminae of the world is provided. It contains 31 valid and 13 invalid species names in four genera with synonyms, collection records, and full literature from 1860's to 2015 for each species. A literature survey of total 477 previous papers was conducted in this work, and 362 of them are newly added to checklist.

## Introduction

Adoncholaiminae Gerlach and Riemann, 1974 is a subfamily of free-living aquatic nematode family Oncholaimidae Filipjev, 1916 (phylum Nematoda). It consists of four genera (Lorenzen 1981a, Lorenzen 1994, Shimada and Kajihara 2014), viz. *Adoncholaimus* Filipjev, 1918, *Admirandus*, Belogurov and Belogurova 1979, *Kreisoncholaimus* Rachor, 1969, and *Meyersia* Hopper, 1967. *Metoncholaimoides* Wieser, 1953 has been placed in the subfamily before, but Shimada and Kajihara (2014) considered the genus as a junior synonym for *Adoncholaimus*. Adoncholaiminae morphologically differs from the other six subfamilies of family Oncholaimidae Filipjev, 1916 in the order Oncholaimida Siddiqi, 1983 and suborder Oncholaimina de Conink, 1965 (c.f. Filipjev 1916, Maggenti 1981, Siddiqi 1983, Smol and Coomans 2006, Hodda 2011, Shimada and Kajihara 2014) by the well developed sperm-storage organ (Demanian system) in the female (Rachor 1969, Belogurov and Belogurova 1977a, Belogurov and Belogurova 1977b, Belogurov and Belogurova 1977c, Belogurov and Belogurova 1977d, Belogurov and Belogurova 1989, Smol and Coomans 2006), however there have been no molecular phylogenetic evidences at present. Nematodes belonging to Adoncholaiminae are found from all continents, from the equatorial tropic to the two poles. Most of them are marine or brackish, and some are freshwater species (Smol and Coomans 2006).

A taxonomist who wants to describe new species must collect original descriptions and redescriptions of all congeners by literature research. Complete lists of all species and subspecies names of the genera are exceedingly helpful for taxonomic studies and biodiversity surveys. Such lists are more useful if any other information of individual species are included. In the field of free-living nematodes, there is a comprehensive reference work "The Bremerhaven Checklist of Aquatic Nematodes" (Gerlach and Riemann 1973, Gerlach and Riemann 1974) covering almost all species names of aquatic nematodes with their synonym list, sampling sites, and references before early 1970's. Although many revisions of the specific genera or higher taxa with species lists and taxonomic keys (e.g. Greenslade and Nicholas 1991, Huang and Cheng 2012, Shimada and Kajihara 2014) have been published since 1974, full literature of them is not available today.

This work aims to provide a checklist of Adoncholaiminae species of the world with their synonymy, distribution, and literature information for potential taxonomists who (will) study nematodes in the subfamily. A literature survey of 31 valid and 13 invalid species in Adoncholaiminae was conducted. More than 400 papers in which species names of Adoncholaiminae are found, not only taxonomic descriptions and collection records but also ecological, morphological, physiological, and DNA works, were completely collected. However, references not satisfying criteria of publication in Articles 8 and 9 of International Code of Zoological Nomenclature (International Commission on Zoological Nomenclature 1999, International Commission on Zoological Nomenclature 2012a, International Commission on Zoological Nomenclature 2012b), e.g. theses, symposium abstracts, and some web articles, are excluded.

Total 485 previous papers (see References) including any species names of Adoncholaiminae was found through a literature survey. Of these references, only 115 were also cited by Gerlach and Riemann (1974) in section of Adoncholaiminae. New 362 papers are added to literature in the present study, containing 293 published after 1973, and 69 were published before 1974 but neglected by them. However, most of the latter were cited in other sections of their checklist.

The names of oceans and seas were used as defined by International Hydrographic Organization (1953)

## Checklists

### Checklist of the subfamily Adoncholaiminae Gerlach and Riemann, 1974 (Nematoda: Oncholaimida: Oncholaimidae) of the world: genera, species, distribution, and reference list for taxonomists and ecologists

#### Adoncholaiminae

Gerlach and Riemann, 1974

Adoncholaiminae Synonym: subtribe Adoncholaimina Gerlach and Riemann, 1974, used first in Belogurov and Belogurova (1978a)
Adoncholaiminae Synonym: subtribe Meyersiina Belogurov and Belogurova, 1978a
Adoncholaiminae Incorrect original spelling: Adoncholaiminini in Belogurov and Belogurova (1978a), Belogurov and Belogurova (1978b), Belogurov and Belogurova (1988), Belogurov and Belogurova (1989)
Adoncholaiminae Incorrect original spelling: Meyersiinini in Belogurov and Belogurova (1978a), Belogurov and Belogurova (1978b), Belogurov and Belogurova (1988), Belogurov and Belogurova (1989)
Adoncholaiminae Type genus: *Adoncholaimus* Filipjev, 1918 =*Metoncholaimoides* Wieser, 1953 [by Shimada and Kajihara (2014)]
Adoncholaiminae Other genera: *Admirandus* Belogurov and Belogurova, 1979; *Kreisoncholaimus* Rachor, 1969; *Meyersia* Hopper, 1967

##### Notes

The latest taxonomic key to genera: Smol and Coomans (2006)

Revisions and reviews of the subfamily: Belogurov and Belogurova 1978a, Belogurov and Belogurova 1978b, Lorenzen 1981a, Belogurov and Belogurova 1988, Belogurov and Belogurova 1989, Lorenzen 1994, Hope 2007, Smol et al. 2014

#### Adoncholaimus

Filipjev, 1918

Adoncholaimus Synonym: *Metoncholaimoides* Wieser, 1953
Adoncholaimus non *Adoncholaimus* sp. in Menzel (1925) (*= Oncholaimus menzeli* Schneider, 1937)
Adoncholaimus nec *Adoncholaimus pristiuris* in Filipjev and Schuurmans Stekhoven (1941) (= incorrect subsequent spelling of *Metoncholaimus pristiurus*?)
Adoncholaimus Etymology: masculine noun, *ad* (Latin, "to") + genus *Oncholaimus* [*όνχος* (Greek, "fang") + *λαιμός* (Greek, "throat")]
Adoncholaimus Type species: *Adoncholaimus fuscus* (Bastian, 1865) Filipjev, 1918
Adoncholaimus Valid 22 species: *A. fuscus*; *A. aralensis* Filipjev, 1924; *A. crassicaudus* Wieser, 1953; *A. daikokuensis* Shimada and Kajihara, 2014; *A. derjugini* (Ssaweljev, 1912) Filipjev, 1918; *A. exoptatus* Belogurova and Belogurov, 1974; *A. fervidus* Kirjanova, 1955; *A. filicauda* (Galtsova, 1976) Shimada and Kajihara, 2014; *A. indicus* (von Linstow, 1907) Filipjev, 1918; *A. islandicus* Kreis, 1963; *A. lepidus* (de Man 1889) Filipjev, 1918; *A. longicaudatus* Paramonov, 1929; *A. longispiculatus* Gagarin and Nguyen, 2007; *A. oxyuroides* (Allgén, 1934) Gerlach and Riemann, 1974; *A. panicus* Cobb, 1930; *A. parvus* Gagarin and Nguyen, 2003; *A. pseudofervidus* Shimada and Kajihara, 2014; *A. punctatus* (Cobb, 1914) Filipjev, 1924; *A. quadriporus* Belogurova and Belogurov, 1974; *A. squalus* (Wieser, 1953) Shimada and Kajihara, 2014; *A. thalassophygas* (de Man, 1876) Filipjev, 1918; *A. ussuriensis* Mordukhovich *et al.*, 2015
Adoncholaimus Invalid 11 species: *A. angustatus* (Cobb, 1981) Filipjev, 1918; *A. austrogeorgiae* Allgén, 1959; *A. bandaensis* Kreis, 1932; *A. chilkensis* (Stewart, 1914) Kreis, 1934; *A. chinensis* Huang and Zhang, 2009; *A. falklandiae* Allgén, 1959; *A. meridionalis* (Kreis, 1932) Rachor, 1969; *A. nudus* (Kreis, 1932) Rachor, 1969; *A. papillatus* Kreis, 1932; *A. skawensis* (Ditlevsen, 1921) Allgén, 1933; *A. taboguillensis* (Allgén, 1947) Wieser, 1953

##### Notes

Original description: Filipjev (1918)

English translation of original description: Filipjev (1968)

The latest taxonomic key to species: Mordukhovich et al. (2015)

Revisions and reviews of the genus: Filipjev (1924), Kreis (1934), Hopper and Cairns (1959), Chitwood (1960), Rachor (1969), Gerlach and Riemann (1974), Belogurov and Belogurova (1977b), Belogurov and Belogurova (1977d), Belogurov and Belogurova (1977c), Belogurov and Belogurova (1977a), Belogurov and Belogurova (1978b), Belogurov and Belogurova (1978a), Platt and Warwick (1983), Belogurov and Belogurova (1988), Belogurov and Belogurova (1989), Keppner and Tarjan (1989), Somerfield and Warwick (1996), Smol and Coomans (2006), Smol et al. (2014), Shimada and Kajihara (2014)

Molecular phylogeny: Litvaitis et al. 2000, Bhadury et al. 2006a, Bhadury et al. 2006b, Sharma et al. 2006, Bhadury et al. 2007, Meldal et al. 2007, Bik et al. 2010a, Bik et al. 2010b

Collection records of unidentified species (*Adoncholaimus* sp.): see Table 1

#### Adoncholaimus fuscus

(Bastian, 1865) Filipjev, 1918

Adoncholaimus fuscus Synonym: *Oncholaimus fuscus* Bastian, 1865
Adoncholaimus fuscus Synonym?: *Adoncholaimus oxyuroides* Allgén, 1934b [by Wieser (1953), not followed by Shimada and Kajihara (2014)]
Adoncholaimus fuscus Etymology: adjective, *fuscus*, -*a*, -*um* (Latin, "dark")

##### Notes

Holotype: unknown

References: see Table 2

#### Adoncholaimus aralensis

Filipjev, 1924

Adoncholaimus aralensis Incorrect subsequent spelling: *Adoncholaimus araliensis* in Kreis (1934)
Adoncholaimus aralensis Etymology: adjective, Aral (type locality) + *ensis*, -*is*, -*e* (Latin, suffix)

##### Notes

Holotype: unknown

References: see Table 3

#### Adoncholaimus crassicaudus

Wieser, 1953

Adoncholaimus crassicaudus Etymology: adjective, *crassus*, -*a*, -*um* (Latin, "thick") + *cauda* (Latin, "tail") + *us*, *a*, *um* (Latin, suffix)

##### Notes

Holotype: unknown

References: see Table 4

#### Adoncholaimus daikokuensis

Shimada and Kajihara, 2014

Adoncholaimus daikokuensis Etymology: adjective, Daikoku (type locality) + *ensis*, -*is*, -*e* (Latin, suffix)

##### Notes

Holotype: ICHUM (formerly ZIHU) 4162, Hokkaido University Museum, Sapporo, Japan

References: see Table 5

#### Adoncholaimus derjugini

(Ssaweljev, 1912) Filipjev, 1924

Adoncholaimus derjugini Synonym: *Oncholaimus derjugini* Ssaweljev, 1912
Adoncholaimus derjugini Etymology: genitive noun, K. Derjugin (person) + *i* (Latin, suffix)

##### Notes

Holotype: unknown

References: see Table 6

#### Adoncholaimus exoptatus

Belogurova and Belogurov, 1974

Adoncholaimus exoptatus Etymology: adjective, *exoptatus*, -*a*, -*um* (Latin, "greatly desired, longed for")?

##### Notes

Holotype: Institute of Marine Biology of the Far Eastern Branch, USSR Academy of Sciences, Vladivostok, USSR

References: see Table 7

#### Adoncholaimus fervidus

Kirjanova, 1955

Adoncholaimus fervidus Etymology: adjective, *fervidus*, *-a*, -*um* (Latin, "fiery")?

##### Notes

Holotype: unknown

References: see Table 8

#### Adoncholaimus filicauda

(Galtsova, 1976) Shimada and Kajihara, 2014

Adoncholaimus filicauda Synonym: *Metoncholaimoides filicauda* Galtsova, 1976
Adoncholaimus filicauda Etymology: appositive noun, *fīlum* (Latin, "thread") + *cauda* (Latin, "tail")

##### Notes

Holotype: Collection No. 10, Institute of Zoology, USSR Academy of Sciences, Leningrad, USSR

References: see Table 9

#### Adoncholaimus indicus

(von Linstow, 1907) Filipjev, 1918

Adoncholaimus indicus Synonym: *Oncholaimus indicus* von Linstow, 1907
Adoncholaimus indicus Etymology: adjective, *indicus*, -*a*, -*um* (Latin, "Indian")

##### Notes

Holotype: unknown

References: see Table 10

#### Adoncholaimus islandicus

Kreis, 1963

Adoncholaimus islandicus Etymology: adjective, *islandicus*, -*a*, -*um* (Latin, "Icelandic")

##### Notes

Holotype: unknown

References: see Table 11

#### Adoncholaimus lepidus

(de Man 1889) Filipjev, 1918

Adoncholaimus lepidus Synonym: *Oncholaimus lepidus* de Man, 1889
Adoncholaimus lepidus non *Oncholaimus lepidus* sensu Schneider (1906): see *Adoncholaimus thalassophygas*
Adoncholaimus lepidus nec *Adoncholaimus lepidus* sensu Warwick and Gage 1975: see *Adoncholaimus panicus*
Adoncholaimus lepidus Synonym?: *Metoncholaimoides filicauda*Wieser 1953 [by Belogurov and Galtsova (1983), not followed by Shimada and Kajihara (2014)]
Adoncholaimus lepidus Etymology: adjective, *lepidus*, -*a*, -*um* (Latin, "agreeable")?

##### Notes

Holotype: unknown

References: see Table 12

#### Adoncholaimus longicaudatus

Paramonov, 1929

Adoncholaimus longicaudatus Etymology: adjective, *longus*, -*a*, -*um* (Latin, "long") + *caudatus*, -*a*, -*um* (Latin, "tailed")

##### Notes

Holotype: unknown

References: see Table 13

#### Adoncholaimus longispiculatus

Gagarin and Nguyen, 2007

Adoncholaimus longispiculatus incorrect subsequent spelling: *Adoncholaimus longicaudatus* in Nguyen and Gagarin (2013a)
Adoncholaimus longispiculatus Etymology: adjective, *longus*, -*a*, -*um* (Latin, "long") + *spiculatus*, -*a*, -*um* (Latin, "spiculed")

##### Notes

Holotype: Institute for Biology of Inland Waters, Russian Academy of Sciences, Borok, Russia

Referemces: see Table 14

#### Adoncholaimus oxyuroides

(Allgén, 1934) Gerlach and Riemann, 1974

Adoncholaimus oxyuroides Synonym: *Oncholaimus oxyuroides* Allgén, 1934
Adoncholaimus oxyuroides Etymology: adjective, *Oncholaimus oxyuris* (species name) + *oides* (Latin, "resembling")

##### Notes

Holotype: unknown

References: see Table 15

#### Adoncholaimus panicus

Cobb, 1930

Adoncholaimus panicus Synonym: *Adoncholaimus lepidus* sensu Warwick and Gage (1975) [by Platt and Warwick (1983)]
Adoncholaimus panicus Etymology: adjective, *panicus*, -*a*, -*um* (Latin, "panicky")?

##### Notes

Holotype: unknown

References: see Table 16

#### Adoncholaimus parvus

Gagarin and Nguyen, 2003

Adoncholaimus parvus Etymology: adjective, *parvus*, -*a*, -*um* (Latin, "panicky")?

##### Notes

Holotype: Institute for Biology of Inland Waters, Russian Academy of Sciences, Borok, Russia

References: see Table 17

#### Adoncholaimus pseudofervidus

Shimada and Kajihara, 2014

Adoncholaimus pseudofervidus Etymology: adjective, *pseudo* (Latin, "fake") + *Adoncholaimus fervidus* (species name)

##### Notes

Holotype: ICHUM (formerly ZIHU) 4184, Hokkaido University Museum, Sapporo, Japan

References: see Table 18

#### Adoncholaimus punctatus

(Cobb, 1914) Filipjev, 1924

Adoncholaimus punctatus Synonym: *Oncholaimus punctatus* Cobb, 1914
Adoncholaimus punctatus Etymology: adjective, *punctatus*, -*a*, -*um* (Latin, "pointed")

##### Notes

Holotype: unknown

References: see Table 19

#### Adoncholaimus quadriporus

Belogurova and Belogurov, 1974

Adoncholaimus quadriporus Etymology: appositive noun, *quadri* (Latin, "four") + *porus* (Latin, "pore")

##### Notes

Holotype: Institute of Marine Biology of the Far Eastern Branch, USSR Academy of Sciences, Vladivostok, USSR

References: see Table 20

#### Adoncholaimus squalus

(Wieser, 1953) Shimada and Kajihara, 2014

Adoncholaimus squalus Synonym: *Metoncholaimoides squalus* Wieser, 1953
Adoncholaimus squalus incorrect subsequent spelling: *Metoncholaimus squalus* in Lorenzen (1976)
Adoncholaimus squalus Etymology: appositive noun, *squalus* (Latin, a kind of shark)?

##### Notes

Holotype: unknown

References: see Table 21

#### Adoncholaimus thalassophygas

(de Man, 1876) Filipjev, 1918

Adoncholaimus thalassophygas Synonym: *Oncholaimus thalassophygas* de Man, 1876
Adoncholaimus thalassophygas Synonym: *Adoncholaimus thalassophygas tvaerminneanus* Schneider, 1926 = *Oncholaimus lepidus* sensu Schneider 1906 [by Filipjev (1929b)]
Adoncholaimus thalassophygas Etymology: appositive noun, *θάλασσα* (Greek, "sea") + *Φυγάς* (Greek, "fugitive")

##### Notes

Holotype: unknown

References: see Table 22

#### Adoncholaimus ussuriensis

Mordukhovich et al., 2015

Adoncholaimus ussuriensis Etymology: adjective, Ussury (type locality) + *ensis*, -*is*, -*e* (Latin, suffix)

##### Notes

Holotype: MN UY 14-2 A1, Zoological Museum of Far Eastern Federal University, Vladivostok, Russia

References: see Table 23

#### Adoncholaimus angustatus

(Cobb, 1891) Filipjev, 1918

Adoncholaimus angustatus Invalid: transferred to the genus *Viscosia* by Kreis (1934)
Adoncholaimus angustatus Synonym: *Oncholaimus angustatus* Cobb, 1891
Adoncholaimus angustatus Etymology: adjective, *angustatus*, -*a*, -*um* (Latin, "narrowed")

##### Notes

Holotype: unknown

References: see Table 24

#### Adoncholaimus austrogeorgiae

Allgén, 1959

Adoncholaimus austrogeorgiae Invalid: treated as a *species inquirenda* by Shimada and Kajihara (2014)
Adoncholaimus austrogeorgiae Etymology: genitive noun, *Austrogeorgia* (Latin, "South Georgia") + *e* (Latin, suffix)

##### Notes

Holotype: unknown

References: see Table 25

#### Adoncholaimus bandaensis

Kreis, 1932

Adoncholaimus bandaensis Invalid: transferred to the genus *Meyersia* by Hopper (1967); see *Meyersia bandaensis*

#### Adoncholaimus chilkensis

(Stewart, 1914) Kreis, 1934

Adoncholaimus chilkensis Invalid: treated as a *species inquirenda* by Shimada and Kajihara (2014)
Adoncholaimus chilkensis Synonym: *Oncholaimus chilkensis* Stewart, 1914
Adoncholaimus chilkensis Etymology: adjective, Chilka (type locality) + *ensis*, -*is*, -*e* (Latin, suffix)

##### Notes

Holotype: No. ZEV. 6237/7, Indian Museum, Kolkata, India

References: see Table 26

#### Adoncholaimus chinensis

Huang and Zhang, 2009

Adoncholaimus chinensis Invalid: synonymized with *Admirandus multicavus* by Mordukhovich et al. (2015); see *Admirandus multicavus*
Adoncholaimus chinensis Etymology: adjective, China (type locality) + *ensis*, -*is*, -*e* (Latin, suffix)

##### Notes

Holotype: LUH 001 (LUL 08801), Institute of Oceanology, Chinese Academy of Sciences, Qingdao, China

#### Adoncholaimus falklandiae

Allgén, 1959

Adoncholaimus falklandiae Invalid: treated as a *species inquirenda* by Shimada and Kajihara (2014)
Adoncholaimus falklandiae Etymology: genitive noun, Falkland (type locality) + *iae* (Latin, suffix)

##### Notes

Holotype: unknown

References: see Table 27

#### Adoncholaimus meridionalis

Kreis, 1932

Adoncholaimus meridionalis Invalid: transferred to the genus *Meyersia* by Hopper 1967; see *Meyersia meridionalis*

#### Adoncholaimus nudus

Kreis, 1932

Adoncholaimus nudus Invalid: transferred to the genus *Kreisoncholaimus* by Rachor (1969); see *Kreisoncholaimus nudus*

#### Adoncholaimus papillatus

Kreis, 1932

Adoncholaimus papillatus Invalid: transferred to the genus *Admirandus* by Shimada and Kajihara (2014); see *Admirandus papillatus*

#### Adoncholaimus skawensis

(Ditlevsen, 1922) Allgén, 1933

Adoncholaimus skawensis Invalid: transferred to the genus *Oncholaimus* again by Allgén (1935b)
Adoncholaimus skawensis Synonym: *Oncholaimus skawensis* Ditlevsen, 1922
Adoncholaimus skawensis Etymology: adjective, Skaw (type locality) + *ensis*, -*is*, -*e* (Latin, suffix)

##### Notes

Holotype: unknown

References: see Table 28

#### Adoncholaimus taboguillensis

(Allgén, 1947) Wieser, 1953

Adoncholaimus taboguillensis Invalid: treated as a *species inquirenda* by Shimada and Kajihara (2014)
Adoncholaimus taboguillensis Synonym: *Viscosia taboguillensis* Allgén, 1947
Adoncholaimus taboguillensis Etymology: adjective, Taboguilla (type locality) + *ensis*, -*is*, -*e* (Latin, suffix)

##### Notes

Holotype: unknown

References: see Table 29

#### Admirandus

Belogurov and Belogurova, 1979

Admirandus Etymology: masculine noun, *admirandus*, -*a*, -*um* (Latin, "remarkable";"wonderful")?
Admirandus Type species: *Admirandus multicavus* Belogurov and Belogurova, 1979
Admirandus Valid three species: *A. multicavus*; *A. belogurovi* Tchesunov *et al*., 2010; *A. papillatus* (Kreis, 1932) Shimada and Kajihara, 2014
Admirandus The genus name was published in Belogurov and Belogurova (1977a), Belogurov and Belogurova (1977d), Belogurov and Belogurova (1978a), Belogurov and Belogurova (1978b) prior to original description, but had been unavailable before Belogurov and Belogurova 1979 [Article 13.1 of International Code of Zoological Nomenclature (International Commission on Zoological Nomenclature 1999)]

##### Notes

Original description: Belogurov and Belogurova (1979)

Revisions and reviews of the genus: Belogurov and Belogurova (1977b), Belogurov and Belogurova (1977a), Belogurov and Belogurova (1978b), Belogurov and Belogurova (1978a), Belogurov and Belogurova (1988), Belogurov and Belogurova (1989), Tchesunov et al. (2010), Smol et al. (2014), Mordukhovich et al. (2015)

#### Admirandus multicavus

Belogurov and Belogurova, 1979

Admirandus multicavus Etymology: appositive noun, *multi* (Latin, "many") + *cavus*, *-a*, -*um* (Latin, "hole")

##### Notes

Holotype: MN-92, Department of Zoology, Far Eastern State University, Vladivostok, USSR

References: see Table 30

#### Admirandus belogurovi

Tchesunov et al., 2010

Admirandus belogurovi Etymology: genitive noun, O.I. Belogurov (person) + *i* (Latin, suffix)

##### Notes

Holotype: Department of Nematology, Institute of Ecology and Biological Resources (IEBR), Vietnam Academy of Science and Technology, Hanoi, Vietnam

References: see Table 31

#### Admirandus papillatus

(Kreis, 1932) Shimada and Kajihara, 2014

Admirandus papillatus Synonym: *Adoncholaimus papillatus* Kreis, 1932
Admirandus papillatus Etymology: adjective, *papillatus*, -*a*, -*um* (Latin, "having papillae")

##### Notes

Holotype: unknown

References: see Table 32

#### Kreisoncholaimus

Rachor, 1969

Kreisoncholaimus Etymology: masculine noun, H.A. Kreis (person) + genus *Oncholaimus*
Kreisoncholaimus Type and only valid species: *Kreisoncholaimus nudus* (Kreis, 1932) Rachor, 1969

##### Notes

Original description: Rachor (1969)

Revisions and reviews of genus: Gerlach and Riemann (1974), Belogurov and Belogurova (1977a), Belogurov and Belogurova (1977d), Belogurov and Belogurova (1978a), Belogurov and Belogurova (1978b), Belogurov and Belogurova (1988), Belogurov and Belogurova (1989), Keppner and Tarjan (1989), Smol and Coomans (2006), Smol et al. (2014)

#### Kreisoncholaimus nudus

(Kreis, 1932) Rachor, 1969

Kreisoncholaimus nudus Synonym: *Adoncholaimus nudus* Kreis, 1932
Kreisoncholaimus nudus Etymology: adjective, *nudus*, -*a*, -*um* (Latin, "naked")

##### Notes

Holotype: unknown

References: see Table 33

#### Meyersia

Hopper, 1967

Meyersia Etymology: feminine noun, S.P. Meyers (person) + *ia* (Latin, prefix)
Meyersia Type species: *Meyersia major* Hopper, 1967
Meyersia Valid five species: *M. major*; *M. bandaensis* (Kreis, 1932) Hopper, 1967; *M. japonica* Yoshimura, 1982; *M. meridionalis* (Kreis, 1932) Hopper, 1967; *M. minor* Hopper, 1967

##### Notes

Original description: Hopper (1967)

Revisions and reviews of genus: Rachor (1969), Gerlach and Riemann (1974), Belogurov and Belogurova (1977b), Belogurov and Belogurova (1977d), Belogurov and Belogurova (1977c), Belogurov and Belogurova (1977a), Belogurov and Belogurova (1978b), Belogurov and Belogurova (1978a), Platt and Warwick (1983), Belogurov and Belogurova (1988), Belogurov and Belogurova (1989), Keppner and Tarjan (1989), Smol and Coomans (2006), Smol et al. (2014)

Collection records of unidentified species (*Meyersia* sp.): see Table 34

#### Meyersia major

Hopper, 1967

Meyersia major Etymology: adjective, *major*, -*or*, -*us* (Latin, "larger")

##### Notes

Holotype: Collection No. 5120, Type slide No. 153, Canadian National Collection of Nematodes, Entomology Research Institute, Ottawa, Canada

References: see Table 35

#### Meyersia bandaensis

(Kreis, 1932) Hopper, 1967

Meyersia bandaensis Synonym: *Adoncholaimus bandaensis* Kreis, 1932
Meyersia bandaensis Etymology: adjective, Banda (type locality) + *ensis*, -*is*, -*e* (Latin, suffix)

##### Notes

Holotype: unknown

References: see Table 36

#### Meyersia japonica

Yoshimura, 1982

Meyersia japonica Etymology: adjective, *japonicus*, -*a*, -*um* (Latin, "Japanese")

##### Notes

Holotype: 790811B-V-4, Seto Marine Biological Laboratory, Wakayama, Japan

References: see Table 37

#### Meyersia meridionalis

(Kreis, 1932) Hopper, 1967

Meyersia meridionalis Synonym: *Adoncholaimus meridionalis* Kreis, 1932
Meyersia meridionalis Etymology: adjective, *meridionalis*, -*is*, -*e* (Latin, "southern")

##### Notes

Holotype: unknown

References: see Table 38

#### Meyersia minor

Hopper, 1967

Meyersia minor Etymology: adjective, *minor*, -*or*, -*us* (Latin, "larger")

##### Notes

Holotype: Collection No. 5122, Type slide No. 154, Canadian National Collection of Nematodes, Entomology Research Institute, Ottawa, Canada

References: see Table 39

#### Metoncholaimoides

Wieser, 1953

Metoncholaimoides Invalid: transferred to the genus *Adoncholaimus* by Shimada and Kajihara (2014); see *Adoncholaimus*
Metoncholaimoides Etymology: *genus Metoncholaimus* + *oides* (Latin, "resembling")
Metoncholaimoides Type species: *Metoncholaimoides squalus* Wieser, 1953

#### Metoncholaimoides squalus

Wieser, 1953

Metoncholaimoides squalus Invalid: transferred to the genus *Adoncholaimus* by Shimada and Kajihara (2014); see *Adoncholaimus squalus*

#### Metoncholaimoides filicauda

Galtsova, 1976

Metoncholaimoides filicauda Invalid: transferred to the genus *Adoncholaimus* by Shimada and Kajihara (2014); see *Adoncholaimus filicauda*

## Supplementary Material

XML Treatment for Adoncholaiminae

XML Treatment for Adoncholaimus

XML Treatment for Adoncholaimus fuscus

XML Treatment for Adoncholaimus aralensis

XML Treatment for Adoncholaimus crassicaudus

XML Treatment for Adoncholaimus daikokuensis

XML Treatment for Adoncholaimus derjugini

XML Treatment for Adoncholaimus exoptatus

XML Treatment for Adoncholaimus fervidus

XML Treatment for Adoncholaimus filicauda

XML Treatment for Adoncholaimus indicus

XML Treatment for Adoncholaimus islandicus

XML Treatment for Adoncholaimus lepidus

XML Treatment for Adoncholaimus longicaudatus

XML Treatment for Adoncholaimus longispiculatus

XML Treatment for Adoncholaimus oxyuroides

XML Treatment for Adoncholaimus panicus

XML Treatment for Adoncholaimus parvus

XML Treatment for Adoncholaimus pseudofervidus

XML Treatment for Adoncholaimus punctatus

XML Treatment for Adoncholaimus quadriporus

XML Treatment for Adoncholaimus squalus

XML Treatment for Adoncholaimus thalassophygas

XML Treatment for Adoncholaimus ussuriensis

XML Treatment for Adoncholaimus angustatus

XML Treatment for Adoncholaimus austrogeorgiae

XML Treatment for Adoncholaimus bandaensis

XML Treatment for Adoncholaimus chilkensis

XML Treatment for Adoncholaimus chinensis

XML Treatment for Adoncholaimus falklandiae

XML Treatment for Adoncholaimus meridionalis

XML Treatment for Adoncholaimus nudus

XML Treatment for Adoncholaimus papillatus

XML Treatment for Adoncholaimus skawensis

XML Treatment for Adoncholaimus taboguillensis

XML Treatment for Admirandus

XML Treatment for Admirandus multicavus

XML Treatment for Admirandus belogurovi

XML Treatment for Admirandus papillatus

XML Treatment for Kreisoncholaimus

XML Treatment for Kreisoncholaimus nudus

XML Treatment for Meyersia

XML Treatment for Meyersia major

XML Treatment for Meyersia bandaensis

XML Treatment for Meyersia japonica

XML Treatment for Meyersia meridionalis

XML Treatment for Meyersia minor

XML Treatment for Metoncholaimoides

XML Treatment for Metoncholaimoides squalus

XML Treatment for Metoncholaimoides filicauda

## Figures and Tables

**Table 1.** References of unidentified species in *Adoncholaimus*. Abbreviations: C, citation; D, description; E, ecology; F, feeding and culture; G, genus diagnosis; L, species list of genus; M, morphology; N, DNA taxonomy; O, other experiment; P, population and fauna; R, taxonomic revision; T, taxonomy.

Reference	Collection			T					E		M	O	C
	Locality	Country	Habitat	D	G	R	L	N	P	F			
Schulz 1935b	Lake Ganzirri, Sicilia Island, Mediterranean Sea	Italy	brackish	×									
Allgén 1942	-	-	-						×				
Riemann 1969	Arensch, Cuxhaven, North Sea	Germany	plants						×				
Ward 1973	Liverpool Bay, Irish Sea	United Kingdom	muddy or clean sand with gravel						×				
Belogurov and Belogurova 1979	Urup, Kuril Islands, North Pacific Ocean	Russia	intertidal	×									
Krishnamurthy et al. 1980	Bay of Bengal	India	mangrove						×				
Subramanian et al. 1984	Vellar to Coleroon estuary, Bay of Bengal	India	mangrove						×				
Aryuthaka 1985 (*Metoncholaimoides* sp.)	Yojiro, Kagoshima Bay, North Pacific Ocean	Japan	sand and seagrasses, 1–3 m deep						×				
Gerdes et al. 1985b	Gavish Sabkha, along Gulf of Elat, Red Sea	Egypt	brackish						×				
Hodda and Nicholas 1985	Hunter estuary, South Pacific Ocean	Australia	intertidal mud with plants						×				
Hammer 1986	-	-	-										×
Lambshead 1986	Bute Island, Firth of Clyde, Irish Sea	United Kingdom	sediment						×				
Alongi 1990	Bowling Green Bay, South Pacific Ocean	Australia	intertidal						×				
Matsumoto et al. 1992	Ariake Sea, Eastern China Sea	Japan	intertidal mud						×				
Ólafsson et al. 1993	off Askö, Baltic Sea	Sweden	34 m deep						×				
Riemann 1995	Porcupine Seabight, North Atlantic Ocean	off Ireland	deep sea sediment						×				
Villano and Warwick 1995	Palude della Rose, Venezia, Adriatic Sea, Mediterranean Sea	Italy	sediment						×				
Blome 1996	Arensch, North Sea	Germany	intertidal						×				
Gee and Somerfield 1997	Sungai Pasir, Malacca Strait	Malaysia	mangrove						×				
Blaxter et al. 1998	-	-	-					×					
Pallo et al. 1998	Gulf of Riga, Baltic Sea	Latvia; Estonia	sediment						×				
Somerfield et al. 1998	Merbok estuary, Malacca Strait	Malaysia	mangrove						×				
Gyedu-Ababio et al. 1999	Swartkops estuary, Algoa Bay, Indian Ocean	South Africa	brackish sediment						×				
Moens et al. 1999a	Schelde estuary, North Sea	The Netherlands	sediment						×				
Moens et al. 1999b	Schelde estuary, North Sea	The Netherlands	sediment						×				
Aarnio 2000	Åland archipelago, Baltic Sea	Finland	subtidal sand						×				
Moens et al. 2000	-	-	-										×
Warwick and Villano 2000	same as Villano and Warwick 1995								×				
Blaxter 2001	-	-	-										×
Gagarin 2001	North East Yakutia, Arctic Sea	Russia	no data						×				
Gheskière et al. 2002	De Panne, North Sea	Belgium	sediment						×				
Gwyther and Fairweather 2002	Barwon estuary, Victoria, Bass Strait	Australia	mud						×				
Wu et al. 2002	Chongming, Shanghai, Eastern China Sea	China	soil with plants						×				
Gwyther 2003	Barwon estuary, Victoria, Bass Strait	Australia	sediment						×				
Ullberg and Ólafsson 2003b	Sandviken, Askö, Baltic Sea	Sweden	sand						×				
Ullberg and Ólafsson 2003a	Sandviken, Askö, Baltic Sea	Sweden	sand						×				
Clarke et al. 2004	-	-	-					×					
Gagarin and Nguyen 2005	Nhue River, Hanoi	Vietnam	sediment						×				
Mullin et al. 2005	no data	-	sediment					×					
Pavlyuk and Trebukhova 2005	Amur Bay, Japan Sea	Russia	6.4–7.5 m deep						×				
Wu et al. 2005	Shanghai, Eastern China Sea	China	soil						×				
Bhadury et al. 2006a	-	-	-					×					
Pavlyuk and Trebukhova 2006	Nha Trang Bay, South China Sea	Vietnam	sediment						×				
Urban-Malinga et al. 2006	Hel Peninsula, Puck Bay, Baltic Sea	Poland	sediment						×				
van Straalen and Roelofs 2006	-	-	-										×
Chen et al. 2007	Yangtze estuary, Shanghai, Eastern China Sea	China	plants						×				
Pavithran et al. 2007	Central Indian Ocean Basin, Indian Ocean	off India	deep sea sediment						×				
Pavlyuk et al. 2007a	Amur Bay, Japan Sea	Russia	intertidal and subtidal sediment						×				
Pavlyuk et al. 2007b	same as Pavlyuk et al. 2007a								×				
Schratzberger et al. 2007	North Sea	off United Kingdom	sediment						×				
Shen et al. 2007	Qingdao, Yellow Sea	China	intertidal sand					×					
Timm et al. 2007	Reigi Bay, Hiiumaa Island, Baltic Sea	Estonia	brackish sediment						×				
Torres-Pratis and Schizas 2007	Magueyes Island, Caribbean Sea	Puerto Rico	mangrove						×				
Quang et al. 2007	Khe Nhan, Can Gio, South China Sea	Vietnam	mud among mangrove						×				
de Leonardis et al. 2008	Manfredonia, Adriatic Sea; Gallipoli, Ionian Sea, Mediterranean Sea	Italy	60–700 m deep						×				
Hose et al. 2008	Lake Martin, Victoria	Australia	freshwater sediment						×				
Ivanova et al. 2008	Tikhaya Lagoon, Amur Bay, Japan Sea	Russia	freshwater and brackish sediment						×				
Pavlyuk and Trebukhova 2008	Amur Bay, Japan Sea	Russia	sediment						×				
Chu et al. 2009	-	-	-					×					
Pavithran et al. 2009	Central Indian Ocean Basin, Indian Ocean	off India	deep sea sediment						×				
van Megen et al. 2009	no data	The Netherlands	soil					×					
Willems et al. 2009	Hättorna, Skagerrak	Sweden	sand, 23–28 m deep						×				
Belogurova 2010	Amur Bay; Kozmino, Japan Sea	Russia	intertidal to subtidal						×				
Bik et al. 2010a	no data	-	no data					×					
Bik et al. 2010b	same as Bik et al. 2010a							×					
Nanajkar and Ingole 2010	Karwar; Ratnagiri, Arabian Sea	India	subtidal sediment						×				
Semprucci et al. 2010	Adriatic Sea, Mediterranean Sea	Italy	sediment						×				
Venekey et al. 2010	no data	Brazil	no data						×				
Gyedu-Ababio 2011	Swartkops estuary, Algoa Bay; Gamtoos estuary, St. Francis Bay, Indian Ocean	South Africa	subtidal sediment						×				
Ngo and Vo 2011	Can Gio, Ho Chi Minh, South China Sea	Vietnam	sediment among mangrove						×				
Pavlyuk and Trebukhova 2011	Jeju Island, Eastern China Sea	South Korea	intertidal sediment						×				
Portnova et al. 2011	Håkon Mosby Mud Volcano, Norwegian Sea	off Norway	1277–1294 m deep						×				
Sawangarreruks et al. 2011	Tha Chin estuary, Gulf of Thailand	Thailand	sediment among mangrove						×				
Tsujino and Uchida 2011	Zigozen, Hiroshima Bay, Seto Inland Sea	Japan	intertidal mud without algae						×				
Fonseca and Fehlauer-Ale 2012	São Sebastião, South Atlantic Ocean	Brazil	intertidal sediment					×					
Nguyen et al. 2012	Mekong River Delta, South China Sea	Vietnam	freshwater sediment						×				
van Straalen and Roelofs 2012	-	-	-										×
Zeppilli et al. 2012	Gulf of Lions, Mediterranean Sea	France	265–434 m deep						×				
Belogurova 2013	Peter the Great Gulf, Japan Sea	Russia	270–800 m deep						×				
Carriço et al. 2013	Saint-Nic, North Atlantic Ocean	France	fine sand						×				
Gingold et al. 2013	El Tornillal, Gulf of California	Mexico	sediment						×				
Ngo and Ngo 2013	Can Gio, Long Hòa, Ho Chi Minh	Vietnam	shrimp growing ponds						×				
Pinto et al. 2013	Itamaraca Island, South Atlantic Ocean	Brazil	sediment among mangrove						×				
Sturhan 2013	Hooge Island, North Sea	Germany	soil						×				
Moens et al. 2014	Veerse Meer, North Sea	The Netherlands	brackish							×			
Ngo et al. 2014	Can Gio, Long Hòa, Ho Chi Minh	Vietnam	sediment among mangrove						×				
Portnova et al. 2014a	Nyegga, Storegga Slide, Norwegian Sea	off Norway	714–739 m deep						×				
Portnova et al. 2014b	same as Portnova et al. 2014b								×				
Zeppilli et al. 2014	Azores Archipelago, North Atlantic Ocean	Portuguese	206–1900 m deep						×				
Pastor de Ward et al. 2015	San Julián Bay, Santa Cruz, South Atlantic Ocean	Argentina	intertidal sediment						×				

**Table 2.** References of *Adoncholaimus fuscus*. Abbreviations: C, citation; D, description; E, ecology; F, feeding and culture; G, genus diagnosis; L, species list of genus; M, morphology; N, DNA taxonomy; O, other experiment; P, population and fauna; R, taxonomic revision; T, taxonomy.

Reference	Collection			T					E		M	O	C
	Locality	Country	Habitat	D	G	R	L	N	P	F			
Bastian 1865 (*O. fuscus*)	Falmouth, English Channel	United Kingdom	mud	×									
Möbius 1873 (*O. fuscus*)	-	-	-										×
Bütschli 1874 (*O. fuscus*)	Kiel Bay, Baltic Sea	Germany	no data								×		
Villot 1875(*O. fuscus*)	-	-	-										×
von Linstow 1878(*O. fuscus*)	-	-	-				×						
de Man 1886(*O. fuscus*)	Schelde estuary, Walcheren, North Sea	The Netherlands	no data	×									
de Man 1888(*O. fuscus*)	-	-	-										×
de Man 1889a(*O. fuscus*)	-	-	-										×
de Man 1889b(*O. fuscus*)	-	-	-						×				
de Man 1889c(*O. fuscus*)	-	-	-										×
von Linstow 1889(*O. fuscus*)	-	-	-				×						
Jägerskiöld 1901(*O. fuscus*)	-	-	-										×
von Linstow 1902(*O. fuscus*)	-	-	-										×
Jägerskiöld 1904(*O. fuscus*)	SW coast of Jutland Peninsula, North Sea	Denmark	no data						×				
Stewart 1906(*O. fuscus*)	-	-	-										×
Horsman 1912(*O. fuscus*)	Ynyslas, Irish Sea	United Kingdom	sand						×				
Stewart 1914b(*O. fuscus*)	-	-	-										×
Filipjev 1918	-	-	-			×							
Ditlevsen 1919(*O. fuscus*)	Snoghøj; Kongebro, Little Belt	Denmark	intertidal to 15 m deep						×				
Allgén 1921(*O. fuscus*)	-	-	-										×
Micoletzky 1921(*O. fuscus*)	-	-	-										×
Filipjev 1924	-	-	-			×							
Allgén 1925(*O. fuscus*)	-	-	-										×
Filipjev 1925	-	-	-				×						
Allgén 1927(*O. fuscus*)	Barsebäckshamn, Øresund	Sweden	algae	×									
Schodduyn 1927(*O. fuscus*)	Slack estuary, near Boulogne-sur-mer, English Channel	France	bottom sediment						×				
Cobb 1930	-	-	-								×		
von Wülker and Schuurmans Stekhoven 1933	-	-	-										×
Allgén 1934b(*O. fuscus*)	-	-	-						×				
Kreis 1934	-	-	-			×							
Allgén 1935a	-	-	-						×				
Schuurmans Stekhoven 1935a	Zuiderzee, North Sea	The Netherlands	no data	×									
Schuurmans Stekhoven 1935b	Nieuport, English Channel; Zuiderzee, North Sea	Belgium; The Netherlands	no data						×				
Schuurmans Stekhoven et al. 1935	Zuiderzee, North Sea	The Netherlands	no data						×				
Schuurmans Stekhoven 1936b	-	-	-						×				
Schulz 1936	Amrum, North Sea	Germany	subtidal sand						×				
Schneider 1939	-	-	-						×				
Bresslau and Schuurmans Stekhoven 1940	Helgoland, North Sea	Germany	no data						×				
de Vos and Redeke 1941	Wadden Sea, North Sea	The Netherlands	no data						×				
Schuurmans Stekhoven 1942a	Øresund	Belgium	no data						×				
Schuurmans Stekhoven 1942b	Yerseke, North Sea	The Netherlands	oyster bed						×				
Schuurmans Stekhoven 1946	Stockholm Archipelago, Baltic Sea	Sweden	no data						×				
Chitwood 1951	Woods Hole, Massachusetts, North Atlantic Ocean	United States	no data						×				
Gerlach 1953b	Kiel Bay, Baltic Sea	Germany	sand						×				
Wieser 1953	-	-	-			×							
Gerlach 1954b	-	-	-						×				
Schuurmans Stekhoven 1954	-	-	-						×				
Remane 1955	-	-	-										×
el Maghraby and Perkins 1956	Wave Crest, Whitstable, North Sea	United Kingdom	mud on slipway						×				
Wieser et al. 1957	Rum Bay, Plymouth, English Channel	United Kingdom	intertidal mud						×				
Gerlach 1958	Kiel Fjord; Vodrups Flak, Kiel Bay, Baltic Sea	Germany	sand; gravel, 2–13 m deep						×				
Capstick 1959	Blyth estuary, North Sea	United Kingdom	sediment						×				
Gerlach 1959	-	-	-						×				
Paramonov 1962(*O. fuscus*)	-	-	-		×								
Kreis 1963	-	-	-										×
de Coninck 1965	-	-	-										×
Bilio 1966	-	-	-						×				
Coles 1966	Skippers Island, Thorpe-le-Soken, Essex, North Sea	United Kingdom	soil among plant roots						×				
Riemann 1966 (A. aff. fuscus)	Cuxhaven-Groden; Ostemündung, North Sea	Germany	sand, intertidal; 13–15 m deep	×									
Filipjev 1968	Schleswig-Holsteinischen, North Sea	Germany	plants in salt marsh						×				
Paramonov 1968(*O. fuscus*)	-	-	-		×								
Hopper 1969	Kingsport, Nova Scotia, North Atlantic Ocean	Canada	blackish-grey mud among plants	×									
Lorenzen 1969	Schleswig-Holsteinischen	Germany	brackish plants in salt marsh						×				
Rachor 1969	same as Rachor 1970										×		
Giere 1970	North Sea	Germany	sand						×				
Rachor 1970	Baltic Sea; North Sea	Germany	no data						×				
Warwick 1971	Lympstone, Exe estuery, English Channel	United Kingdom	sand						×				
Hope and Murphy 1972	-	-	-				×						
Hope 1974	-	-	-										×
Gerlach and Riemann 1974	-	-	-				×						
Warwick and Gage 1975	Loch Etive, Scotland	United Kingdom	surface						×				
Belogurov and Belogurova 1977a	-	-	-								×		
Belogurov and Belogurova 1977b	-	-	-								×		
Belogurov and Belogurova 1978a	-	-	-			×							
Belogurov and Belogurova 1978b	-	-	-			×							
Belogurova 1978	Itrup, Kuril Islands, North Pacific Ocean	Russia	subtidal	×									
Belogurov and Belogurova 1979	-	-	-										×
Belogurova and Belogurov 1979	Kuril Islands, North Pacific Ocean	Russia	10 m deep						×				
Calcoen and de Kegel 1979	Nieuwpoort-Bad, North Sea	Belgium	sand								×		
Maertens and Coomans 1979	-	-	-										×
Calcoen and de Kegel 1980	-	-	-										×
Sherman and Coull 1980	Crabhaul Creek, North Inlet, North Atlantic Ocean	United States	mud with detritus						×				
Bouwman 1981	Ems estuary, North Sea	Germany	intertidal sandy sediment	×									
Blome 1982	Sylt Island, North Sea	Germany	sand						×				
Field et al. 1982	Exe estuary, English Channel	United Kingdom	mud, sand						×				
Yoshimura 1982	-	-	-										×
Bouwman 1983	same as Bouwman 1981								×				
Chabaud et al. 1983	-	-	-								×		
Heip et al. 1983	-	-	-						×				
Platt and Warwick 1983	no data	United Kingdom	no data	×									
Gerdes and Krumbein 1984	-	-	-						×				
Belogurov 1985	-	-	-								×		
Gerdes et al. 1985a	Mellum Island, North Sea	Germany	sand						×				
Gerdes et al. 1985b (*O. fuscus*)	Gavish Sabkha, along Gulf of Elat, Red Sea	Egypt	brackish						×				
Heip et al. 1985	-	-	-						×				
Belogurov and Belogurova 1988	-	-	-			×							
Coomans et al. 1988	-	-	-										×
Belogurov and Belogurova 1989	Far East	Russia	no data			×							
Furstenberg and Vincx 1989	-	-	-										×
Bussau 1990a	Wadden Sea, North Sea	Germany	sand						×				
Bussau 1990b	same as Bussau 1990a			×									
Kennish 1990	-	-	-						×				
Mohd Long 1990	Forth estuary, Scotland, North Sea	United Kingdom	mud						×				
Trett and Feil 1990	-	-	-						×				
Alkemade et al. 1993	Stroodorpepolder, North Sea	The Netherlands	brackish, plants leaves						×				
Clarke 1993	same as Warwick 1971								×				
Soetaert et al. 1994	Western Scheldt, North Sea	The Netherlands	sediment						×				
Kennedy 1995	Exe estuary, English Channel	United Kingdom	sand						×				
Soetaert et al. 1995	Ems estuary; Somme; Western Scheldt, North Sea	Germany; France; Belgium	brackish sediment						×				
Blome 1996	Borkum; Mellum; Ems estuary; Weser estuary, North Sea	Germany	intertidal sand; microbial mats						×				
Blome and Faubel 1996	off Kaiser-Wuhelm-Koog, Elbe estuary, North Sea	Germany	sand with mud and shells						×				
Moens and Vincx 1997	Western Scheldt, North Sea	The Netherlands	subtidal to intertidal, sand and mud							×			
Platt and Ball 1997	-	-	-						×				
Travizi and Vidaković 1997	Mirna estuary; Adriatic Sea, Mediterranean Sea	Croatia	silt and sand						×				
Yushin and Malakhov 1997	-	-	-										×
Attrill 1998	-	-	-						×				
Craig and Ashman 1998	Solway Firth, Irish Sea	United Kingdom	intertidal						×				
Moens and Vincx 1998	same as Moens and Vincx 1997									×			
Justine and Jamieson 1999	-	-	-										×
Moens et al. 1999b	Western Scheldt, North Sea	The Netherlands	mud and coarse sand							×			
Moens et al. 1999c	Western Scheldt, North Sea	The Netherlands	intertidal mud							×			
Tita et al. 1999	-	-	-							×			
Moens et al. 2000	Molenplaat; Paulina salt marsh, Schelde estuary, North Sea	The Netherlands	intertidal							×			
Litvaitis et al. 2000	Paulina salt marsh, Schelde estuary, North Sea	The Netherlands	silty sediment					×					
de Smet et al. 2001	-	-	-						×				
Justine 2002	-	-	-										×
Moens et al. 2002	Paulina salt marsh, Schelde estuary, North Sea	The Netherlands	brackish, lower vegetation zone							×			
Tita et al. 2002	Parc du Bic, St. Lawrence estuary, Gulf of St. Lawrence	Canada	mud and sand						×				
Yushin et al. 2002	-	-	-										×
Bilgrami and Gaugler 2004	-	-	-										×
Bilgrami and Gaugler 2005	-	-	-										×
Cook et al. 2005	Southend-on-Sea or Southampton, English Channel	United Kingdom	sediment					×					
Kathiresan and Qasim 2005	Andaman and Nicobar Islands, Indian Ocean	India	mangrove						×				
Moens et al. 2005a	Paulina salt marsh, Schelde estuary, North Sea	The Netherlands	sediment							×			
Moens et al. 2005b	Western Scheldt, North Sea	The Netherlands	no data							×			
Afanasiev-Grigoriev et al. 2006	-	-	-										×
Bhadury et al. 2006a	England	United Kingdom	no data					×					
Bhadury et al. 2006b	same as Bhadury et al. 2006a							×					
Haseman 2006	-	-	-										×
Krassen 2006	-	-	-										×
Sharma et al. 2006	Aransas Pass, Port Aransas, North Atlantic Ocean	United States	sediment					×					
Smol and Coomans 2006	-	-	-		×								
Bhadury et al. 2007	Tamar estuary, English Channel	United Kingdom	no data					×					
Chinnadurai and Fernando 2007	Vellar estuary, Bay of Bengal	India	sediment among mangrove						×				
Meldal et al. 2007	Southampton Water, English Channel	United Kingdom	intertidal sediment					×					
Fonseca and Gallucci 2008	-	-	-										×
Moodley et al. 2008	Westerschelde estuary, North Sea	The Netherlands	mud						×				
Semprucci et al. 2008	-	-	-						×				
Arigó et al. 2009	Akrotiri Bay, Mediterranean Sea	Cyprus	sediment						×				
Giere 2009	-	-	-										×
Huang and Zhang 2009	-	-	-										×
Leduc 2009	-	-	-										×
Bik et al. 2010a	no data	-	no data					×					
Vandepitte et al. 2010	-	-	-						×				
dos Santos and Moens 2011	-	-	-										×
Sminova and Fadeeva 2011	Japan Sea	Russia	sandy sediment						×				
Shokoohi et al. 2013	-	-	-					×					
Shoshin 2013	-	-	-						×				
Gibson and Fuentes 2014	-	-	-					×					
Smol et al. 2014	-	-	-		×								
Shimada and Kajihara 2014	-	-	-			×							
Mordukhovich et al. 2015	-	-	-			×							

**Table 3.** References of *Adoncholaimus aralensis*. Abbreviations: C, citation; D, description; E, ecology; F, feeding and culture; G, genus diagnosis; L, species list of genus; M, morphology; N, DNA taxonomy; O, other experiment; P, population and fauna; R, taxonomic revision; T, taxonomy.

Reference	Collection			T					E		M	O	C
	Locality	Country	Habitat	D	G	R	L	N	P	F			
Filipjev 1924	Aralsk landing bridge, Aral Sea	Kazakhstan	brackish	×									
Kreis 1934 (*A. araliensis*)	-	-	-			×							
Schneider 1937	-	-	-										×
Rachor 1969	-	-	-										×
Gerlach and Riemann 1974	-	-	-				×						
Tchesunov 1976	Caspian Sea	USSR	brackish						×				
Gagarin 1978	Astrakhan	Russia	freshwater						×				
Belogurov and Belogurova 1979	-	-	-										×
Belogurova and Belogurov 1979	Urup, Kuril Islands, North Pacific Ocean	Russia	subtidal						×				
Mordukhai-Boltovskoi 1979	Caspian Sea	USSR	brackish						×				
Tsalolikhin 1979	Lake Issyk-Kul	Kyrgyzstan	brackish						×				
Gagarin 1981	-	-	-										×
Tsalolikhin 1982	-	-	-										×
Tchesunov 1983	Caspian Sea	-	brackish						×				
Belogurov and Belogurova 1988	-	-	-			×							
Gagarin and Nguyen 2003	-	-	-										×
Smol and Coomans 2006	-	-	-		×								
Mokievsky 2009	-	-	-										×
Gusakov and Gagarin 2012a	Lake El'ton	Russia	brackish						×				
Gusakov and Gagarin 2012b	same as Gusakov and Gagarin 2012a								×				
Shoshin 2013	-	-	-						×				
Shimada and Kajihara 2014	-	-	-			×							
Mordukhovich et al. 2015	-	-	-			×							

**Table 4.** References of *Adoncholaimus crassicaudus*. Abbreviations: C, citation; D, description; E, ecology; F, feeding and culture; G, genus diagnosis; I, Fixation method; L, species list of genus; M, morphology; N, DNA taxonomy; O, other experiment; P, population and fauna; R, taxonomic revision; T, taxonomy.

Reference	Collection			T					E		M	O	C
	Locality	Country	Habitat	D	G	R	L	N	P	F			
Wieser 1953	San Antonio, Valparais, South Pacific Ocean	Chile	intertidal, filamentous green algae	×									
Mawson 1958	Kerguelen Islands, Indian Ocean; Macquarie Island, South Pacific Ocean	French Southern and Antarctic Lands	intertidal to 69 m deep, rock; green algae; seaweeds	×									
Wieser 1959	same as Wieser 1953								×				
Rachor 1969	-	-	-										×
Timm and Hackney 1969	Bodega Bay, California	United States	algae									I	
Gerlach and Riemann 1974	-	-	-				×						
Belogurov and Belogurova 1979	Hopeful Bay, Kerguelen Islands, Indian Ocean	French Southern and Antarctic Lands	silty sand, 3–30 m deep	×									
Belogurov and Belogurova 1988	-	-	-			×							
Greenslade 1989	-	-	-						×				
Ingels et al. 2014	-	-	-						×				
Shimada and Kajihara 2014	-	-	-			×							
Mordukhovich et al. 2015	-	-	-			×							

**Table 5.** References of *Adoncholaimus daikokuensis*. Abbreviations: C, citation; D, description; E, ecology; F, feeding and culture; G, genus diagnosis; L, species list of genus; M, morphology; N, DNA taxonomy; O, other experiment; P, population and fauna; R, taxonomic revision; T, taxonomy.

Reference	Collection			T					E		M	O	C
	Locality	Country	Habitat	D	G	R	L	N	P	F			
Shimada and Kajihara 2014	Daikoku Island, Hokkaido, North Pacific Ocean	Japan	intertidal mud in seagrass bed	×									
Mordukhovich et al. 2015	-	-	-			×							

**Table 6.** References of *Adoncholaimus derjugini*. Abbreviations: C, citation; D, description; E, ecology; F, feeding and culture; G, genus diagnosis; L, species list of genus; M, morphology; N, DNA taxonomy; O, other experiment; P, population and fauna; R, taxonomic revision; T, taxonomy.

Reference	Collection			T					E		M	O	C
	Locality	Country	Habitat	D	G	R	L	N	P	F			
Ssaweljev 1912 (*O. derjugini*)	Mogil'noye Bay, Kildin Island, Barents Sea	Russia	no data	×									
Filipjev 1924	-	-	-			×							
Filipjev 1925	-	-	-						×				
Kreis 1934	-	-	-			×							
Allgén 1957	-	-	-						×				
Rachor 1969	-	-	-										×
Gerlach and Riemann 1974	-	-	-				×						
de Smet et al. 2001	-	-	-						×				
Shimada and Kajihara 2014	-	-	-			×							
Mordukhovich et al. 2015	-	-	-			×							

**Table 7.** References of *Adoncholaimus exoptatus*. Abbreviations: C, citation; D, description; E, ecology; F, feeding and culture; G, genus diagnosis; L, species list of genus; M, morphology; N, DNA taxonomy; O, other experiment; P, population and fauna; R, taxonomic revision; T, taxonomy.

Reference	Collection			T					E		M	O	C
	Locality	Country	Habitat	D	G	R	L	N	P	F			
Belogurova and Belogurov 1974	Itrup, Kuril Islands, North Pacific Ocean	Russia	rhizoids of subtidal algae	×									
Belogurov and Belogurova 1977a	-	-	-								×		
Belogurov and Belogurova 1977d	-	-	-								×		
Belogurov and Belogurova 1979	-	-	-										×
Belogurova and Belogurov 1979	Kuril Islands, North Pacific Ocean	Russia	subtidal to intertidal, sand among seagrasses and stones	×									
Smolyanko and Belogyrov 1987	-	-	-										×
Belogurov and Belogurova 1988	-	-	-			×							
Belogurov and Belogurova 1989	Far East	Russia	no data			×							
Shoshin 2013	-	-	-						×				
Smol et al. 2014	-	-	-		×								
Shimada and Kajihara 2014	-	-	-			×							
Mordukhovich et al. 2015	-	-	-			×							

**Table 8.** References of *Adoncholaimus fervidus*. Abbreviations: C, citation; D, description; E, ecology; F, feeding and culture; G, genus diagnosis; L, species list of genus; M, morphology; N, DNA taxonomy; O, other experiment; P, population and fauna; R, taxonomic revision; T, taxonomy.

Reference	Collection			T					E		M	O	C
	Locality	Country	Habitat	D	G	R	L	N	P	F			
Kirjanova 1955	Japan Sea	Russia	among algae	×									
Kirjanova 1966	same as Kirjanova 1955			×									
Belogurova and Belogurov 1974	Iturup, Kuril Islands, North Pacific Ocean	Russia	subtidal, sand among barnacles and algal rhizoids	×									
Belogurov and Belogurova 1977a	-	-	-								×		
Belogurov and Belogurova 1977d	-	-	-								×		
Belogurov and Belogurova 1979	Kuril Islands, North Pacific Ocean	Russia	subtidal mudflat	×									
Belogurova and Belogurov 1979	Kuril Islands, North Pacific Ocean	Russia	supratidal to subtidal, sand among algae, barnacles, and polychaetes	×									
Smolyanko and Belogyrov 1987	-	-	-										×
Belogurov and Belogurova 1988	-	-	-			×							
Belogurov and Belogurova 1989	Far East	Russia	no data			×							
Pavlyuk et al. 2007a	Amur Bay, Japan Sea	Russia	intertidal						×				
Pavlyuk et al. 2007b	same as Pavlyuk et al. 2007a								×				
Ivanova et al. 2008	Amur Bay, Japan Sea	Russia	intertidal						×				
Pavlyuk et al. 2009	Peter the Great Bay, Japan Sea	Russia	30 m deep						×				
Belogurova 2010	Amur Bay; Kozmino, Japan Sea	Russia	intertidal to subtidal						×				
Zvyagintsev and Moshchenko 2010	Vladivostok	Russia	cooling system of power station						×				
Shoshin 2013	-	-	-						×				
Shimada and Kajihara 2014	-	-	-			×							
Mordukhovich et al. 2015	-	-	-			×							

**Table 9.** References of *Adoncholaimus filicauda*. Abbreviations: C, citation; D, description; E, ecology; F, feeding and culture; G, genus diagnosis; L, species list of genus; M, morphology; N, DNA taxonomy; O, other experiment; P, population and fauna; R, taxonomic revision; T, taxonomy.

Reference	Collection			T					E		M	O	C
	Locality	Country	Habitat	D	G	R	L	N	P	F			
Galtsova 1976 (*M. filicauda*)	White Sea	Russia	intertidal silty sand	×									
Belogurov and Galtsova 1983	-	-	-										×
Galtsova 1985 (*M. filicauda*)	same as Galtsova 1976			×									
Shimada and Kajihara 2014	-	-	-			×							
Mordukhovich et al. 2015	-	-	-			×							

**Table 10.** References of *Adoncholaimus indicus*. Abbreviations: C, citation; D, description; E, ecology; F, feeding and culture; G, genus diagnosis; L, species list of genus; M, morphology; N, DNA taxonomy; O, other experiment; P, population and fauna; R, taxonomic revision; T, taxonomy.

Reference	Collection			T					E		M	O	C
	Locality	Country	Habitat	D	G	R	L	N	P	F			
von Linstow 1907 (*O. indicus*)	Conning, Matla, Bay of Bengal	India	brackish	×									
Stewart 1914a (*O. indicus*)	Conning, Matla, Bay of Bengal	India	brackish	×									
Stewart 1914b (*O. indicus*)	-	-	-										×
Filipjev 1918	-	-	-			×							
Filipjev 1924	-	-	-			×							
Menzel 1925	-	-	-										×
Allgén 1934c	-	-	-										×
Kreis 1934	-	-	-			×							
Schneider 1937 (*O. indicus*)	-	-	-										×
Filipjev 1968	-	-	-			×							
Gerlach and Riemann 1974	-	-	-				×						
Kathiresan and Qasim 2005 (*O. indicus*)	-	-	-						×				
Shimada and Kajihara 2014	-	-	-			×							
Mordukhovich et al. 2015	-	-	-			×							

**Table 11.** References of *Adoncholaimus islandicus*. Abbreviations: C, citation; D, description; E, ecology; F, feeding and culture; G, genus diagnosis; L, species list of genus; M, morphology; N, DNA taxonomy; O, other experiment; P, population and fauna; R, taxonomic revision; T, taxonomy.

Reference	Collection			T					E		M	O	C
	Locality	Country	Habitat	D	G	R	L	N	P	F			
Kreis 1963	Eyja Fjord, Greenland Sea	Iceland	mud	×									
Rachor 1969	-	-	-										×
Gerlach and Riemann 1974	-	-	-				×						
Belogurov and Belogurova 1977a	-	-	-								×		
Belogurov and Belogurova 1977d	-	-	-								×		
Belogurov and Belogurova 1978a	-	-	-			×							
Belogurov and Belogurova 1978b	-	-	-			×							
Belogurov and Belogurova 1979	-	-	-										×
Alekseyev and Dizendorf 1981	near Aniva Bay, Sakhalin, Sea of Okhotsk	Russia	mud with detritus, 0.6 m deep	×									
Furstenberg and Vincx 1989	-	-	-										×
de Smet et al. 2001	-	-	-						×				
Smol and Coomans 2006	-	-	-		×								
Shimada and Kajihara 2014	-	-	-			×							
Mordukhovich et al. 2015	-	-	-			×							

**Table 12.** References of *Adoncholaimus lepidus*. Abbreviations: C, citation; D, description; E, ecology; F, feeding and culture; G, genus diagnosis; L, species list of genus; M, morphology; N, DNA taxonomy; O, other experiment; P, population and fauna; R, taxonomic revision; T, taxonomy; V, developmental biology.

Reference	Collection			T					E		M	O	C
	Locality	Country	Habitat	D	G	R	L	N	P	F			
de Man 1889b (*O. lepidus*)	Walcheren, North Sea	The Netherlands	brackish soil among plant roots	×									
von Linstow 1889(*O. lepidus*)	-	-	-				×						
Steiner 1917(*O. lepidus*)	-	-	-										×
Filipjev 1918	-	-	-			×							
Micoletzky 1921(*O. lepidus*)	-	-	-		×								
de Man 1922b(*O. lepidus*)	-	-	-						×				
Steiner 1922(*O. lepidus*)	-	-	-										×
Filipjev 1924	-	-	-			×							
Allgén 1929b(*O. lepidus*)	-	-	-										×
Allgén 1929c(*O. lepidus*)	-	-	-										×
Allgén 1933(*O. lepidus*)	-	-	-										×
de Coninck and Schuurmans Stekhoven 1933	-	-	-										×
von Wülker and Schuurmans Stekhoven 1933	-	-	-										×
Allgén 1934c(*O. lepidus*)	-	-	-										×
Kreis 1934	-	-	-			×							
Schulz 1935a(*O. lepidus*)	Kiel Bay, Baltic Sea (in Gerlach and Riemann 1974)	Germany	coastal groundwater						×				
Schuurmans Stekhoven 1935a	Zuiderzee, North Sea	The Netherlands	no data	×									
Schuurmans Stekhoven et al. 1935	Zuiderzee, North Sea	The Netherlands	no data						×				
Schuurmans Stekhoven 1936b	-	-	-						×				
Schneider 1939	-	-	-						×				
de Coninck 1943	Cape Reykjanes, North Atlantic Ocean	Iceland	wet terrestrial soil near brackish pond						×				
de Coninck 1944	same as de Coninck 1943			×									
Gerlach 1949	Kiel Bay, Baltic Sea	Germany	otoplana zone and wet sand						×				
Goodey 1951(*O. lepidus*)	-	-	-										×
Timm 1952	Chesapeake Bay, North Atlantic Ocean	United States	no data	×									
Gerlach 1953a	Tvärminne, Gulf of Finland, Baltic Sea	Finland	otoplana zone; groundwater	×									
Gerlach 1953b	Kiel Bay, Baltic Sea	Germany	sand						×				
Wieser 1953	-	-	-			×							
Gerlach 1954a	-	-	-						×				
Gerlach 1954b	-	-	-						×				
Gerlach 1954c	-	-	-						×				
Brinck et al. 1955	Simrishamn Beach, Scania, Baltic Sea	Sweden	coastal groundwater						×				
Delamare Deboutteville et al. 1955	-	-	-						×				
Gerlach 1958	-	-	-						×				
Remane and Schlieper 1958	-	-	-						×				
Gerlach 1959	-	-	-						×				
Delamare Deboutteville 1960	-	-	-										×
Paramonov 1962(*O. lepidus*)	-	-	-		×								
Goodey and 1963(*O. lepidus*)	-	-	-		×								
Bilio 1966	-	-	-						×				
Bilio 1967	-	-	-						×				
Filipjev 1968	-	-	-			×							
Paramonov 1968 (*O. lepidus*)	-	-	-		×								
Lorenzen 1969	Schleswig-Holsteinischen	Germany	plants in brackish salt marsh						×				
Rachor 1969	same as Rachor 1970										×		
Rachor 1970	Kiel Bay, Baltic Sea	Germany	groundwater, sand and gravel						×				
Remane and Schlieper 1971	-	-	-										×
Gerlach and Riemann 1974	-	-	-				×						
Aminova and Galtsova 1978	Chupa, Kandalaksha Gulf, White Sea	Russia	30–40 m deep						×				
Belogurov and Galtsova 1983	White Sea	Russia	no data	×									
Heip et al. 1983	-	-	-						×				
Platt and Warwick 1983	-	-	-										×
Belogurov and Belogurova 1988	-	-	-			×							
Belogurov and Belogurova 1989	Far East	Russia	no data			×							
Furstenberg and Vincx 1989	-	-	-										×
Voronov and Panchin 1995	Kandalaksha Gulf, White Sea	Russia	near White Sea Biological Station									V	
Platt and Ball 1997	-	-	-						×				
de Smet et al. 2001	-	-	-						×				
Gagarin and Nguyen 2003	-	-	-										×
Smol and Coomans 2006	-	-	-		×								
Shimada and Kajihara 2014	-	-	-			×							
Mordukhovich et al. 2015	-	-	-			×							

**Table 13.** References of *Adoncholaimus longicaudatus*. Abbreviations: C, citation; D, description; E, ecology; F, feeding and culture; G, genus diagnosis; L, species list of genus; M, morphology; N, DNA taxonomy; O, other experiment; P, population and fauna; R, taxonomic revision; T, taxonomy.

Reference	Collection			T					E		M	O	C
	Locality	Country	Habitat	D	G	R	L	N	P	F			
Paramonov 1929	Dnieper-Bug estuary; Domakhy, Black Sea	Ukraine	5.3–6.4 m deep	×									
Gerlach and Riemann 1974	-	-	-				×						
Shimada and Kajihara 2014	-	-	-			×							
Mordukhovich et al. 2015	-	-	-			×							

**Table 14.** References of *Adoncholaimus longispiculatus*. Abbreviations: C, citation; D, description; E, ecology; F, feeding and culture; G, genus diagnosis; L, species list of genus; M, morphology; N, DNA taxonomy; O, other experiment; P, population and fauna; R, taxonomic revision; T, taxonomy.

Reference	Collection			T					E		M	O	C
	Locality	Country	Habitat	D	G	R	L	N	P	F			
Gagarin and Nguyen 2007	Mekong River Delta, Kiu Long River, Ham Luong	Vietnam	shore lake sediment among mangrove, 1.2 m deep	×									
Nguyen and Gagarin 2013a	-	-	-										×
Nguyen and Gagarin 2013b (*A. longicaudatus*)	-	-	-										×
Smol et al. 2014	-	-	-		×								
Shimada and Kajihara 2014	-	-	-			×							
Mordukhovich et al. 2015	-	-	-			×							

**Table 15.** References of *Adoncholaimus oxyuroides*. Abbreviations: C, citation; D, description; E, ecology; F, feeding and culture; G, genus diagnosis; L, species list of genus; M, morphology; N, DNA taxonomy; O, other experiment; P, population and fauna; R, taxonomic revision; T, taxonomy.

Reference	Collection			T					E		M	O	C
	Locality	Country	Habitat	D	G	R	L	N	P	F			
Allgén 1934c (*O. oxyuroides*)	Trelleborg to Ystad, Baltic Sea	Sweden	brackish plant roots	×									
Allgén 1950a (*O. oxyuroides*)	Bondhålet, Baltic Sea	Sweden	brackish plant roots	×									
Wieser 1953	-	-	-										×
Gerlach and Riemann 1974	-	-	-				×						
Shimada and Kajihara 2014	-	-	-			×							
Mordukhovich et al. 2015	-	-	-			×							

**Table 16.** References of *Adoncholaimus panicus*. Abbreviations: C, citation; D, description; E, ecology; F, feeding and culture; G, genus diagnosis; L, species list of genus; M, morphology; N, DNA taxonomy; O, other experiment; P, population and fauna; R, taxonomic revision; T, taxonomy.

Reference	Collection			T					E		M	O	C
	Locality	Country	Habitat	D	G	R	L	N	P	F			
Cobb 1930	no data (Massachusetts? in Gerlach and Riemann 1974)	-	no data	×									
Chitwood 1951	Woods Hole, Massachusetts, North Atlantic Ocean	United States	no data						×				
Kreis 1934	-	-	-			×							
Dollfus 1946	-	-	-										×
Rachor 1969	same as Rachor 1970										×		
Rachor 1970	Schilksee, Kiel Bay, Baltic Sea	Germany	sand and gravel, 20–50 cm deep from surface	×									
Gerlach and Riemann 1974	-	-	-				×						
Warwick and Gage 1975 (*A. lepidus*)	Loch Etive, Scotland	United Kingdom	0–2 m deep						×				
Belogurov and Belogurova 1977a	-	-	-								×		
Belogurov and Belogurova 1977d	-	-	-								×		
Belogurov and Belogurova 1978a	-	-	-			×							
Belogurov and Belogurova 1978b	-	-	-			×							
Belogurov and Belogurova 1979	-	-	-										×
Chabaud et al. 1983	-	-	-								×		
Heip et al. 1983	-	-	-						×				
Platt and Warwick 1983	same as Warwick and Gage 1975			×									
Belogurov and Belogurova 1988	-	-	-			×							
Belogurov and Belogurova 1989	Far East	Russia	no data			×							
Furstenberg and Vincx 1989	-	-	-			×							
Platt and Ball 1997	-	-	-						×				
de Smet et al. 2001	-	-	-						×				
Shimada and Kajihara 2014	-	-	-			×							
Mordukhovich et al. 2015	-	-	-			×							

**Table 17.** References of *Adoncholaimus parvus*. Abbreviations: C, citation; D, description; E, ecology; F, feeding and culture; G, genus diagnosis; L, species list of genus; M, morphology; N, DNA taxonomy; O, other experiment; P, population and fauna; R, taxonomic revision; T, taxonomy.

Reference	Collection			T					E		M	O	C
	Locality	Country	Habitat	D	G	R	L	N	P	F			
Gagarin and Nguyen 2003	Nhue River, Hanoi	Vietnam	freshwater, 1.0–2.5 m deep	×									
Gagarin and Nguyen 2008a	Red River Delta, Bat Lat	Vietnam	brackish silt, 1.0–1.5 m deep						×				
Gagarin and Nguyen 2008b	Chu River, Thanh Hoa	Vietnam	brackish silt, 0.5–1.5 m deep										
Gagarin and Nguyen 2008c	same as Gagarin and Nguyen 2008a								×				
Gagarin and Nguyen 2008d	same as Gagarin and Nguyen 2008b								×				
Gagarin and Nguyen 2012a	Tra Ly River, Thai Bình	Vietnam	sand, 0.5 m deep						×				
Gagarin and Nguyen 2012b	same as Gagarin and Nguyen 2012a								×				
Nguyen and Gagarin 2013a	-	-	-										×
Nguyen and Gagarin 2013b	-	-	-										×
Smol et al. 2014	-	-	-		×								
Shimada and Kajihara 2014	-	-	-			×							
Mordukhovich et al. 2015	-	-	-			×							

**Table 18.** References of *Adoncholaimus pseudofervidus*. Abbreviations: C, citation; D, description; E, ecology; F, feeding and culture; G, genus diagnosis; L, species list of genus; M, morphology; N, DNA taxonomy; O, other experiment; P, population and fauna; R, taxonomic revision; T, taxonomy.

Reference	Collection			T					E		M	O	C
	Locality	Country	Habitat	D	G	R	L	N	P	F			
Shimada and Kajihara 2014	Mukawa, Hokkaido, North Pacific Ocean	Japan	intertidal sessile bivalves	×									
Mordukhovich et al. 2015	-	-	-			×							

**Table 19.** References of *Adoncholaimus punctatus*. Abbreviations: C, citation; D, description; E, ecology; F, feeding and culture; G, genus diagnosis; L, species list of genus; M, morphology; N, DNA taxonomy; O, other experiment; P, population and fauna; R, taxonomic revision; T, taxonomy.

Reference	Collection			T					E		M	O	C
	Locality	Country	Habitat	D	G	R	L	N	P	F			
Cobb 1914 (*O. punctatus*)	Cape Breton Island, North Atlantic Ocean	Canada	freshwater ponds	×									
Steiner 1917 (*O. punctatus*)	-	-	-										×
Cobb 1918 (*O. punctatus*)	-	-	-		×								
Micoletzky 1921 (*O. punctatus*)	-	-	-		×								
Filipjev 1924	-	-	-			×							
Menzel 1925	-	-	-										×
Imamura 1931 (*O. punctatus*)	Komaba, Tokyo	Japan	terrestrial soil						×				
Kreis 1934	-	-	-			×							
Chitwood 1951	-	-	-						×				
Goodey 1951 (*O. punctatus*)	-	-	-										×
Wieser 1953	-	-	-			×							
Paramonov 1962	-	-	-										×
Paramonov 1968	-	-	-										×
Rachor 1969	-	-	-										×
Gerlach and Riemann 1974	-	-	-				×						
Tsalolikhin 1982	-	-	-										×
Esser and Buckingham 1987 (*O. punctatus*)	-	-	-						×				
Smol and Coomans 2006	-	-	-		×								
Shimada and Kajihara 2014	-	-	-			×							
Mordukhovich et al. 2015	-	-	-			×							

**Table 20.** References of *Adoncholaimus quadriporus*. Abbreviations: C, citation; D, description; E, ecology; F, feeding and culture; G, genus diagnosis; L, species list of genus; M, morphology; N, DNA taxonomy; O, other experiment; P, population and fauna; R, taxonomic revision; T, taxonomy.

Reference	Collection			T					E		M	O	C
	Locality	Country	Habitat	D	G	R	L	N	P	F			
Belogurova and Belogurov 1974	Itrup, Kuril Islands, North Pacific Ocean	Russia	intertidal sand among barnacles	×									
Belogurov and Belogurova 1977a	-	-	-								×		
Belogurov and Belogurova 1977d	-	-	-								×		
Belogurov and Belogurova 1978a	-	-	-			×							
Belogurov and Belogurova 1978b	-	-	-			×							
Belogurov and Belogurova 1979	-	-	-										×
Belogurova and Belogurov 1979	Kuril Islands, North Pacific Ocean	Russia	subtidal to intertidal, mud, sand, barnacles, polychaetes, and rhizoids of algae						×				
Belogurov and Belogurova 1988	-	-	-			×							
Belogurov and Belogurova 1989	Far East	Russia	no data			×							
Shoshin 2013	-	-	-						×				
Smol et al. 2014	-	-	-		×								
Shimada and Kajihara 2014	-	-	-			×							
Mordukhovich et al. 2015	-	-	-			×							

**Table 21.** References of *Adoncholaimus squalus*. Abbreviations: C, citation; D, description; E, ecology; F, feeding and culture; G, genus diagnosis; L, species list of genus; M, morphology; N, DNA taxonomy; O, other experiment; P, population and fauna; R, taxonomic revision; T, taxonomy.

Reference	Collection			T					E		M	O	C
	Locality	Country	Habitat	D	G	R	L	N	P	F			
Wieser 1953 (*M. squalus*)	Punta Santa María; Canal FitzRoy, Strait of Magellan	Chile	intertidal sheltered algae and stones with rich sediment	×									
Mawson 1958 (*M. squalus*)	Kerguelen Islands, Indian Ocean; Macquarie Island, South Pacific Ocean	French Southern and Antarctic Lands	intertidal mussel bank	×									
Wieser 1958 (*M. squalus*)	-	-	-						×				
Wieser 1959 (*M. squalus*)	same as Wieser 1953								×				
Hope and Murphy 1972 (*M. squalus*)	-	-	-				×						
Gerlach and Riemann 1974 (*M. squalus*)	-	-	-				×						
Galtsova 1976 (*M. squalus*)	-	-	-										×
Lorenzen 1976 (*M. squalus*)	-	-	-										×
Belogurov and Belogurova 1978a (*M. squalus*)	-	-	-			×							
Belogurov and Belogurova 1978b (*M. squalus*)	-	-	-			×							
Galtsova 1985 (*M. squalus*)	-	-	-										×
Belogurov and Belogurova 1989(*M. squalus*)	Far East	Russia	no data			×							
Greenslade 1989 (*M. squalus*)	-	-	-						×				
Pastor de Ward 1993 (*M. squalus*)	Deseado estuary, Santa Cruz, South Atlantic Ocean	Argentina	intertidal algae	×									
Pastor de Ward 1998 (*M. squalus*)	Deseado estuary, Santa Cruz, South Atlantic Ocean	Argentina	algae						×				
Smol and Coomans 2006 (*M. squalus*)	-	-	-		×								
Ingels et al. 2014 (*M. squalus*)	-	-	-						×				
Smol et al. 2014 (*M. squalus*)	-	-	-		×								
Shimada and Kajihara 2014	-	-	-			×							
Mordukhovich et al. 2015	-	-	-			×							

**Table 22.** References of *Adoncholaimus thalassophygas*. Abbreviations: C, citation; D, description; E, ecology; F, feeding and culture; G, genus diagnosis; L, species list of genus; M, morphology; N, DNA taxonomy; O, other experiment; P, population and fauna; R, taxonomic revision; S, physiology; T, taxonomy.

Reference	Collection			T					E		M	O	C
	Locality	Country	Habitat	D	G	R	L	N	P	F			
de Man 1876b (*O. thalassophygas*)	Walcheren, Middelburg, North Sea	The Netherlands	brackish soil among plant roots	×									
de Man 1876a(*O. thalassophygas*)	-	-	-										×
de Man 1881(*O. thalassophygas*)	Walcheren, Middelburg, North Sea	The Netherlands	brackish soil among plant roots	×									
de Man 1884(*O. thalassophygas*)	same as de Man 1881			×									
de Man 1886 (*O. thalassophygas*)	-	-	-		×								
de Man 1889b(*O. thalassophygas*)	Walcheren, Middelburg, North Sea	The Netherlands	brackish soil among plant roots	×									
von Linstow 1889(*O. thalassophygas*)	-	-	-										×
von Linstow 1890(*O. thalassophygas*)	-	-	-										×
Schneider 1906(*O. lepidus*, *O. thalassophygas*)	Tvärminne, Gulf of Finland, Baltic Sea	Finland	brackish	×									
Ditlevsen 1911(*O. thalassophygas*)	Hellerup, Øresund	Denmark	salt marsh plant roots						×				
Vanhöffen 1917(*O. thalassophygas*)	Vistula Lagoon, Baltic Sea	Prussia	brackish						×				
Filipjev 1918	-	-	-			×							
de Man 1919(*O. thalassophygas*)	same as de Man 1884			×									
Micoletzky 1921(*O. thalassophygas*)	-	-	-		×								
de Man 1922a(*O. thalassophygas*)	Urk, Zuiderzee, North Sea	The Netherlands	no data	×									
Steiner 1922(*O. thalassophygas*)	-	-	-										×
Filipjev 1924	-	-	-			×							
Schneider 1925a(*O. thalassophygas*)	Oldesloe, Holstein	Germany	saltwater spring						×				
Schneider 1925b(*O. thalassophygas*)	-	-	-						×				
Riech 1926(*O. thalassophygas*)	-	-	-										×
Schneider 1926 (*A. t. tvaerminneanus*)	Tvärminne, Finland Bay, Baltic Sea	Finland	brackish	×									
Allgén 1927(*O. thalassophygas*)	Barsebäck; Pilhaken, Øresund	Sweden	mud and green algae; 8–20 m deep	×									
Ditlevsen 1927(*O. thalassophygas*)	-	-	-										×
Riech 1927(*O. thalassophygas*)	-	-	-										×
Schneider 1927a(*A. t. tvaerminneanus*)	same as Schneider 1926								×				
Schneider 1927b (*A. t. tvaerminneanus*)	same as Schneider 1926			×									
de Man 1928	Caen, Ouistreham, English Channel	France	brackish	×									
Allgén 1929a	same as Allgén 1929b								×				
Allgén 1929b(*O. thalassophygas*)	Hälsingborg, Øresund	Sweden	mud, plant roots, and algae	×									
Allgén 1929c(*O. thalassophygas*)	-	-	-										×
Filipjev 1929a	Grande Izhora, Gulf of Finland, Baltic Sea	Russia	brackish	×									
Filipjev 1929b	-	-	-						×				
de Coninck 1930	Assels, Drongen; Zwyn, Knokke	Belgium	soil						×				
Schuurmans Stekhoven 1930	-	-	-						×				
Allgén 1931	-	-	-						×				
de Coninck 1931	-	-	-						×				
Schuurmans Stekhoven 1931	Huiden, Zuiderzee, North Sea	The Netherlands	marine and brackish						×				
Schulz 1932	Kiel Bay, Baltic Sea	Germany	brackish sand						×				
Allgén 1933	Trondheims Fjord, Norwegian Sea	Norway	coastal						×				
de Coninck and Schuurmans Stekhoven 1933	Øresund	Belgium	brackish						×				
Schuurmans Stekhoven 1933	-	-	-						×				
Sick 1933(*O. thalassophygas*)	Steiner Bay, Kiel Bay, Baltic Sea	Germany	brackish detritus and algae						×				
von Wülker and Schuurmans Stekhoven 1933 (*A. t. tvaerminneanus*)	-	-	-										×
Allgén 1934a	Skälderviken,Skåne, Kattegat	Sweden	brackish algae						×				
Allgén 1934b	Hallands Väderö; Torekow, Skåne, Kattegat	Sweden	among plants, 0.5 m deep; shallow water seaweeds						×				
Allgén 1934c	Trelleborg to Ystad, Baltic Sea	Sweden	brackish plant roots						×				
Kreis 1934	-	-	-			×							
Remane 1934	-	-	-						×				
Schuurmans Stekhoven 1934	Lemster or Urk, Zuiderzee, North Sea	The Netherlands	brackish						×				
Allgén 1935a	Øresund	Sweden	cray, sand, stones, shells, sessile bivalves, plants and algae, 2–30 m deep						×				
Allgén 1935b	Föhr Island, North Sea	Germany	no data						×				
Schuurmans Stekhoven 1935a	Zuiderzee, North Sea	The Netherlands	no data						×				
Schuurmans Stekhoven 1935b	Zeebrugge, North Sea	Belgium	no data	×									
Schuurmans Stekhoven et al. 1935	Zuiderzee, North Sea	The Netherlands	no data						×				
Otto 1936	Kiel Bay, Baltic Sea	Germany	no data						×				
Schulz 1936	Amrum, North Sea	Germany	sand						×				
Schuurmans Stekhoven 1936a	-	-	-						×				
Schuurmans Stekhoven 1936b	-	-	-						×				
Schulz 1938	Amrum, North Sea	Germany	sand						×				
Meuche 1939	Lake Sehlendorfer; Lake Waterneverstorfer	Germany	brackish						×				
Schneider 1939	-	-	-						×				
Bresslau and Schuurmans Stekhoven 1940	Heligoland, North Sea	Germany	archiannelids and algae	×									
de Vos and Redeke 1941	-	-	-						×				
Filipjev and Schuurmans Stekhoven 1941	-	-	-										×
Schuurmans Stekhoven 1942a	Øresund	Belgium	no data						×				
Allgén 1947a	-	-	-										×
Allgén 1947b	Hallands Väderö; Torekov, Kattegat	Sweden	algae and seagrass roots						×				
Allgén 1947c	-	-	-										×
Allgén 1949	Balestrand, Norwegian Sea	Norway	brackish						×				
Gerlach 1949	Kiel Bay, Baltic Sea	Germany	sand, 1–100 m deep; otoplana zone						×				
Allgén 1950b	Trondheims Fjord, Norwegian Sea	Norway	algae, seagrasses, and shell sand						×				
Goodey 1951(*O. thalassophygas*)	-	-	-	×									
Allgén 1953	Kristineberg to Fiskebäckskil, Skagerrak	Sweden	seagrasses						×				
Gerlach 1953a	Gulf of Finland, Baltic Sea	Finland	sand, 1–1.5 m deep; otoplana zone	×									
Gerlach 1953b	North Sea; Baltic Sea; Selker Noor	Germany	sand; brackish sediment						×				
Wieser 1953	-	-	-			×							
Gerlach 1954b	-	-	-						×				
Gerlach 1954c	-	-	-						×				
Meyl 1954	Barnstorf	Germany	salt spring						×				
Schuurmans Stekhoven 1954	-	-	-						×				
Allgén 1955	-	-	-						×				
Meyl 1955	Barnstorf; Sülldorf	Germany	salt spring	×									
Paetzold 1955	-	-	-						×				
Schütz and Kinne 1955	Kiel Channel	Germany	brackish						×				
el Maghraby and Perkins 1956	Wave Crest, Whitstable, North Sea	United Kingdom	mud on slipway						×				
Gerlach 1958	Flüggesand; Kieler Fjord, Baltic Sea	Germany	sand, 3–7 m deep						×				
Paetzold 1958	Langen Rieth, Numburg	Germany	salt spring						×				
Remane and Schlieper 1958	-	-	-						×				
Allgén 1959	Cumberland, South Atlantic Ocean	South Georgia Islands	subtidal sediment with stones and algae						×				
Capstick 1959	Blyth estuary, North Sea	United Kingdom	sediment						×				
Gerlach 1959	-	-	-						×				
Coles 1960	Skippers Island, Thorpe le Soken, Essex, North Sea	United Kingdom	brackish soil; marine mud						×				
Loof and Oostenbrink 1961	-	-	-						×				
Schütz 1963 (*A. thalassophygas*)	-	-	-						×				
de Coninck 1965	-	-	-						×				
Bilio 1966	-	-	-						×				
Coles 1966	Skippers Island, Thorpe-le-Soken, Essex, North Sea	United Kingdom	mud						×				
Riemann 1966	Brunsbüttel, Elbe estuary, North Sea	Germany	no data						×				
Schütz 1966 (*A. thalassophygas*)	-	-	-						×				
Andrássy 1967	-	-	-						×				
Bilio 1967	-	-	-						×				
von Thun 1967	-	-	-										×
Filipjev 1968	-	-	-			×							
Lorenzen 1969	Meldorf, North Sea	Germany	no data						×				
Rachor 1969	same as Rachor 1970										×		
Gysels 1970	-	-	-						×				
Rachor 1970	Weser River, Bremerhaven, North Sea	Germany	bank						×				
Gerlach 1971	-	-	-							×			
Gerlach and Schrage 1971	-	-	-										×
Remane and Schlieper 1971	-	-	-						×				
Skoolmun and Gerlach 1971	Weser estuary, North Sea	Germany	brackish sand						×				
Warwick 1971	Exe estuary, English Channel	United Kingdom	mud						×				
Gysels 1972b	Groot Cat, Waasland, Overslag	Belgium	creak						×				
Gysels 1972a	same as Gysels 1972b								×				
Ernst and Goerke 1974	-	-	-									S	
Gerlach and Riemann 1974	-	-	-				×						
Goerke and Ernst 1975	North Sea	Germany	deep sea sediment									S	
Warwick and Gage 1975	Loch Etive, Scotland	United Kingdom	0–2 m deep						×				
Wildish 1976	-	-	-									S	
Gerlach 1977	Nivå, Øresund	Denmark	no data						×				
Kinne 1977	-	-	-							×			
Andrássy 1978	-	-	-						×				
Lopez et al. 1979	Weser estuary, Bremerhaven, North Sea	Germany	intertidal diatom film, brackish							×			
Zaika and Makarova 1979	-	-	-							×			
Karl 1980	-	-	-									S	
Tsalolikhin 1980 (*A. talassophygas*)	Koporye, Gulf of Finland, Baltic Sea	Russia	no data						×				
Bouwman 1981	-	-	-						×				
Lorenzen 1981a	-	-	-			×							
Lorenzen 1981b	Tvärminne, Gulf of Finland, Baltic Sea	Finland	brackish sand								×		
Field et al. 1982	Exe estuary, English Channel	United Kingdom	mud, sand						×				
Tsalolikhin 1982	-	-	-										×
van Meel 1982	-	-	-						×				
Bouwman 1983	Ems estuary, North Sea	The Netherlands	sediment						×				
Heip et al. 1983	-	-	-						×				
Platt and Warwick 1983	-	-	-	×									
Romeyn and Bouwman 1983	Ems estuary, North Sea	The Netherlands	no data							×			
Schiemer et al. 1983	Gulf of Bothnia, Baltic Sea	Finland	sand, 5 m deep; mud, 81 m deep						×				
Bouwman et al. 1984	Nieuwe Statenzijl, Ems estuary, North Sea	The Netherlands	mud						×				
Jensen 1984	Gulf of Finland, Baltic Sea	Finland	subtidal						×				
Eskin and Palmer 1985 (A. cf. thalassophygas)	North Inlet estuary, South Carolina, North Pacific Ocean	United States	mud without vegetation						×				
Heip et al. 1985	-	-	-						×				
Nuss 1985	-	-	-							×			
Vranken et al. 1985	-	-	-										×
Coomans and Heyns 1986	-	-	-										×
Little 1986	Swanpool, Falmouth, English Channel	United Kingdom	brackish lagoon						×				
Vranken and Heip 1986a	-	-	-										×
Vranken and Heip 1986b	-	-	-										×
Jensen 1987	-	-	-							×			
Moore 1987	Firth of Forth, Scotland, North Sea	United Kingdom	brackish						×				
Lopez 1988	-	-	-										×
Prein 1988	-	-	-										×
Riemann and Schrage 1988	Weser estuary, Bremerhaven, North Sea	Germany	intertidal mud							×			
Coomans 1989	-	-	-						×				
Furstenberg and Vincx 1989	-	-	-										×
Riemann et al. 1990	Bremerhaven, North Sea	Germany	intertidal mud, brackish							×			
Trett and Feil 1990	-	-	-						×				
Bongers et al. 1991	-	-	-										×
Hellwig-Armonies et al. 1991	-	-	-										×
Clarke 1993	same as Warwick 1971								×				
Warwick 1993	-	-	-										×
Essink and Romeyn 1994	Dollard, Westerwoldse Aa River, North Sea	The Netherlands	intertidal sand and mud						×				
Lorenzen 1994	-	-	-			×							
Kennedy 1995	Exe estuary, English Channel	United Kingdom	mud						×				
Soetaert et al. 1995	Gironde, Bay of Biscay	France	subtidal sediment						×				
Somerfield et al. 1995	-	-	-										×
Vopel and Arit 1995	Hiddensee Island, Baltic Sea	Germany	floating cyanobacterial mats, brackish						×				
Blome 1996	Ems estuary; Dollart; Weser estuary, North Sea	Germany	intertidal sand						×				
Blome and Faubel 1996	Brokdorf; Außendeich; Mühlenstraßen; Kaiser-Wuhelm-Koog, North Sea	Germany	sand and mud						×				
Jensen 1996	-	-	-										×
Moens and Vincx 1997	Western Scheldt estuary, North Sea	The Netherlands	subtidal to high tide level, sand and mud						×				
Ólafsson and Elmgren 1997	off Askö, Baltic Sea	Sweden	37 m deep						×				
Platt and Ball 1997	-	-	-						×				
Attrill 1998	-	-	-						×				
Essink and Keidel 1998	Westerwoldse Aa River, North Sea	The Netherlands	intertidal sand and mud						×				
Perry and Aumann 1998	-	-	-										×
Moens et al. 1999b	-	-	-										×
Moens et al. 1999d	-	-	-										×
de Smet et al. 2001	-	-	-						×				
Höss et al. 2001	-	-	-										×
Tchesunov 2001	-	-	-										×
Lee 2002	-	-	-										×
Haitzer et al. 2002	-	-	-										×
Riemann and Helmke 2002	Weser estuary, Bremerhaven, North Sea	Germany	intertidal, brackish							×			
Wetzel et al. 2002	Fährinsel Island, Baltic Sea	Germany	macroalgal mats						×				
Essink 2003	-	-	-										×
Steinberg 2003	-	-	-										×
Höckelmann et al. 2004	-	-	-										×
Robinson 2004	-	-	-										×
Arroyo et al. 2006	Åland Archipelago, Baltic Sea	Finland	subtidal sand						×				
Moens et al. 2006	-	-	-										×
O'Halloran et al. 2006	-	-	-										×
Smol and Coomans 2006	-	-	-		×								
Filip and Claus 2008	-	-	-										×
Arigó et al. 2009	Akrotiri Bay, Mediterranean Sea	Cyprus	sediment						×				
Vandepitte et al. 2010	-	-	-						×				
Ólafsson et al. 2013	Åland Archipelago, Baltic Sea	Finland	fine sediment with algae						×				
Urban-Malinga et al. 2013	Vistula Lagoon, Baltic Sea	Poland and Russia	brackish, 27 m deep						×				
Ingels et al. 2014	-	-	-						×				
Kooijman 2014	-	-	-										×
Shimada and Kajihara 2014	-	-	-			×							
Urban-Malinga et al. 2014	Puck Bay, Baltic Sea	Poland	0.7–0.8 m deep						×				
Boufahja et al. 2015	-	-	-										×
Hannachi et al. 2015(*O. thalassophygas*)	Wadi Henna	Tunisia	freshwater sediment						×				
Mordukhovich et al. 2015	-	-	-			×							

**Table 23.** References of *Adoncholaimus ussuriensis*. Abbreviations: C, citation; D, description; E, ecology; F, feeding and culture; G, genus diagnosis; L, species list of genus; M, morphology; N, DNA taxonomy; O, other experiment; P, population and fauna; R, taxonomic revision; T, taxonomy.

Reference	Collection			T					E		M	O	C
	Locality	Country	Habitat	D	G	R	L	N	P	F			
Mordukhovich et al. 2015	Ussury Bay, Peter the Great Bay, Japan Sea	Russia	sand in subtidal seagrass bed	×									

**Table 24.** References of *Adoncholaimus angustatus*. Abbreviations: C, citation; D, description; E, ecology; F, feeding and culture; G, genus diagnosis; L, species list of genus; M, morphology; N, DNA taxonomy; O, other experiment; P, population and fauna; R, taxonomic revision; T, taxonomy

Reference	Collection			T					E		M	O	C
Cobb 1891	Locality	Country	Habitat	D	G	R	L	N	P	F			
Cobb 1891 (*O. angustatus*)	Indian Ocean	Arabia	sand	×									
Filipjev 1918	-	-	-			×							

**Table 25.** References of *Adoncholaimus austrogeorgiae*. Abbreviations: C, citation; D, description; E, ecology; F, feeding and culture; G, genus diagnosis; L, species list of genus; M, morphology; N, DNA taxonomy; O, other experiment; P, population and fauna; R, taxonomic revision; T, taxonomy.

Reference	Collection			T					E		M	O	C
	Locality	Country	Habitat	D	G	R	L	N	P	F			
Allgén 1959	Grytviken, South Georgia Island, South Atlantic Ocean	South Georgia and the South Sandwich Islands	kelp rhizoids	×									
Gerlach and Riemann 1974	-	-	-				×						
Ingels et al. 2014	-	-	-						×				
Shimada and Kajihara 2014	-	-	-						×				

**Table 26.** References of *Adoncholaimus chilkensis*. Abbreviations: C, citation; D, description; E, ecology; F, feeding and culture; G, genus diagnosis; L, species list of genus; M, morphology; N, DNA taxonomy; O, other experiment; P, population and fauna; R, taxonomic revision; T, taxonomy.

Reference	Collection			T					E		M	O	C
	Locality	Country	Habitat	D	G	R	L	N	P	F			
Stewart 1914b (*O. chilkensis*)	Chilka Lake, Madras, Bay of Bengal	India	filamentous algae	×									
Annandale 1915(*O. chilkensis*)	Chilka Lake, Madras, Bay of Bengal	India	sponges						×				
Menzel 1925(*O. chilkensis*)	-	-	-										×
Kreis 1932(*O. chilkensis*)	-	-	-										×
Allgén 1934c(*O. chilkensis*)	-	-	-										×
Kreis 1934	-	-	-			×							
Baylis 1936(*O. chilkensis*)	-	-	-	×									
Panikkar and Aiyar 1937(*O. chilkensis*)	-	-	-						×				
Rachor 1969	-	-	-										×
Gerlach and Riemann 1974	-	-	-				×						
Belogurov et al. 1980(*O. chilkensis*)	-	-	-										×
Tsalolikhin 1982	-	-	-										×
Shimada and Kajihara 2014	-	-	-			×							

**Table 27.** References of *Adoncholaimus falklandiae*. Abbreviations: C, citation; D, description; E, ecology; F, feeding and culture; G, genus diagnosis; L, species list of genus; M, morphology; N, DNA taxonomy; O, other experiment; P, population and fauna; R, taxonomic revision; T, taxonomy.

Reference	Collection			T					E		M	O	C
	Locality	Country	Habitat	D	G	R	L	N	P	F			
Allgén 1959	Port William, East Falkland, South Atlantic Ocean	Falkland Islands	sand and algae, 40 m deep	×									
Gerlach and Riemann 1974	-	-	-				×						
Ingels et al. 2014	-	-	-						×				
Shimada and Kajihara 2014	-	-	-			×							

**Table 28.** References of *Adoncholaimus skawensis*. Abbreviations: C, citation; D, description; E, ecology; F, feeding and culture; G, genus diagnosis; L, species list of genus; M, morphology; N, DNA taxonomy; O, other experiment; P, population and fauna; R, taxonomic revision; T, taxonomy

Reference	Collection			T					E		M	O	C
	Locality	Country	Habitat	D	G	R	L	N	P	F			
Ditlevsen 1922 (*O. skawensis*)	Skaw, Skagen, Skagerrak or Kattegat	Denmark	algae, hydroids, and stones	×									
Allgén 1933	Munkholmen; Tautra, Trondheim	Norway	8–60 m deep						×				

**Table 29.** References of *Adoncholaimus taboguillensis*. Abbreviations: C, citation; D, description; E, ecology; F, feeding and culture; G, genus diagnosis; L, species list of genus; M, morphology; N, DNA taxonomy; O, other experiment; P, population and fauna; R, taxonomic revision; T, taxonomy.

Reference	Collection			T					E		M	O	C
	Locality	Country	Habitat	D	G	R	L	N	P	F			
Allgén 1947a (*V. taboguillensis*)	Taboguilla Island, Bay of Panama, North Pacific Ocean	Panama	coral	×									
Allgén 1951 (*V. taboguillensis*)	Contadora Island, Bay of Panama, North Pacific Ocean	Panama	sand and stone, 8–10 m deep	×									
Wieser 1953	-	-	-			×							
Gerlach and Riemann 1974	-	-	-				×						
Shimada and Kajihara 2014	-	-	-			×							

**Table 30.** References of *Admirandus multicavus*. Abbreviations: C, citation; D, description; E, ecology; F, feeding and culture; G, genus diagnosis; L, species list of genus; M, morphology; N, DNA taxonomy; O, other experiment; P, population and fauna; R, taxonomic revision; T, taxonomy.

Reference	Collection			T					E		M	O	C
	Locality	Country	Habitat	D	G	R	L	N	P	F			
Belogurov and Belogurova 1978a	-	-	-			×							
Belogurov and Belogurova 1978b	-	-	-			×							
Belogurov and Belogurova 1979	Slavyanka, Japan Sea	Russia	sand and gravel in lagoon	×									
Belogurov and Belogurova 1988	-	-	-			×							
Belogurov and Belogurova 1989	Far East	Russia	no data			×							
Pavlyuk et al. 2007a	Amur Bay, Japan Sea	Russia	intertidal						×				
Pavlyuk et al. 2007b	same as Pavlyuk et al. 2007a								×				
Ivanova et al. 2008	Amur Bay, Japan Sea	Russia	brackish sediment						×				
Pavlyuk and Trebukhova 2008	Amur Bay, Japan Sea	Russia	subtidal sediment						×				
Huang and Zhang 2009 (*A. chinensis*)	Lianyungang to Rizhao, Yellow Sea	China	intertidal muddy sand	×									
Ivanova et al. 2009	Russky Island, Japan Sea	Russia	intertidal						×				
Belogurova 2010	Amur Bay; Kozmino; Russky Island, Japan Sea	Russia	intertidal to subtidal						×				
Tchesunov et al. 2010	-	-	-										×
Shoshin 2013	-	-	-						×				
Smol et al. 2014	-	-	-		×								
Shimada and Kajihara 2014 (*A. chinensis*)	-	-	-			×							
Mordukhovich et al. 2015	-	-	-	×									

**Table 31.** References of *Admirandus belogurovi*. Abbreviations: C, citation; D, description; E, ecology; F, feeding and culture; G, genus diagnosis; L, species list of genus; M, morphology; N, DNA taxonomy; O, other experiment; P, population and fauna; R, taxonomic revision; T, taxonomy.

Reference	Collection			T					E		M	O	C
	Locality	Country	Habitat	D	G	R	L	N	P	F			
Tchesunov et al. 2010	Be estuary, Nha Trang Bay, South China Sea	Vietnam	intertidal silty sand among mangrove	×									
Mokievsky et al. 2011a	Be estuary, Nha Trang Bay, South China Sea	Vietnam	intertidal silty sand among mangrove						×				
Mokievsky et al. 2011b	same as Mokievsky et al. 2011a								×				
Smol et al. 2014	-	-	-		×								
Mordukhovich et al. 2015	-	-	-			×							

**Table 32.** References of *Admirandus papillatus*. Abbreviations: C, citation; D, description; E, ecology; F, feeding and culture; G, genus diagnosis; L, species list of genus; M, morphology; N, DNA taxonomy; O, other experiment; P, population and fauna; R, taxonomic revision; T, taxonomy.

Reference	Collection			T					E		M	O	C
	Locality	Country	Habitat	D	G	R	L	N	P	F			
Kreis 1932 (*A. papillatus*)	Batavia, Java Sea; Saparua Island, Banda Sea (in Micoletzky and Kreis 1930)	Indonesia	mud; algae in rocky shore	×									
Kreis 1934 (*A. papillatus*)	same as Kreis 1932			×									
Gerlach and Riemann 1974 (*A. papillatus*)	-	-	-				×						
Furstenberg and Vincx 1989 (*A. papillatus*)	Sundays estuary, Indian Ocean	South Africa	brackish, intertidal	×									
Smol and Coomans 2006 (*A. papillatus*)	-	-	-		×								
Shimada and Kajihara 2014	-	-	-			×							

**Table 33.** References of *Kreisoncholaimus nudus*. Abbreviations: C, citation; D, description; E, ecology; F, feeding and culture; G, genus diagnosis; L, species list of genus; M, morphology; N, DNA taxonomy; O, other experiment; P, population and fauna; R, taxonomic revision; T, taxonomy.

Reference	Collection			T					E		M	O	C
	Locality	Country	Habitat	D	G	R	L	N	P	F			
Kreis 1932 (*A. nudus*)	Saparua Island, Banda Sea (in Micoletzky and Kreis 1930)	Indonesia	algae in rocky shore	×									
Kreis 1934(*A. nudus*)	same as Kreis 1932			×									
Rachor 1969	-	-	-			×							
Rachor 1970	-	-	-			×							
Hope and Murphy 1972	-	-	-				×						
Gerlach and Riemann 1974	-	-	-				×						
Belogurov and Belogurova 1978a	-	-	-			×							
Belogurov and Belogurova 1978b	-	-	-			×							
Chabaud et al. 1983	-	-	-								×		
Belogurov and Belogurova 1988	-	-	-			×							
Smol and Coomans 2006	-	-	-		×								
Smol et al. 2014	-	-	-		×								

**Table 34.** References of unidentified species in *Meyersia*. Abbreviations: C, citation; D, description; E, ecology; F, feeding and culture; G, genus diagnosis; L, species list of genus; M, morphology; N, DNA taxonomy; O, other experiment; P, population and fauna; R, taxonomic revision; T, taxonomy.

Reference	Collection			T					E		M	O	C
	Locality	Country	Habitat	D	G	R	L	N	P	F			
Gourbault et al. 1995	Moorea Island, South Pacific Ocean	French Polynesia	intertidal coral sand						×				
Villano and Warwick 1995	Palude della Rose, Adriatic Sea, Mediterranean Sea	Italy	sediment						×				
Warwick and Villano 2000	same as Villano and Warwick 1995								×				
Mirto et al. 2003	Gaeta Gulf, Tyrrhenian Sea, Mediterranean Sea	Italy	fish-farm cage						×				
Ullberg and Ólafsson 2003b	Sandviken, Askö, Baltic Sea	Sweden	sand						×				
Haseman 2006	Hausgarten, off Svalbard, Fram Strait	off Norway	deep sea sand with marl and silt						×				
Pavlyuk and Trebukhova 2006	Nha Trang Bay, South China Sea	Vietnam	sediment						×				
Liu et al. 2008	Victoria Harbour, Hong Kong, South China Sea	China	5–23 m deep						×				
Sougawa et al. 2008	Manazuru Port, Sagami Bay, North Pacific Ocean	Japan	5–6 m deep						×				
Bik et al. 2010a (*Meyersia* sp. or *Metaparoncholaimus* sp.)	off Crozet Islands, Indian Ocean	French Southern and Antarctic Lands	deep sea sediment					×					
Bik et al. 2010b (*Meyersia* sp. or *Metaparoncholaimus* sp.)	same as Bik et al. 2010a							×					
Ingole and Singh 2010	off Larsemann Hills, Prydz Bay, Indian Ocean	Antarctica	525–722 m deep						×				
Nanajkar et al. 2010	Zuari estusry, Goa, Arabian Sea	India	clayey sediment, 9 m deep						×				
Venekey et al. 2010	no data	Brazil	no data						×				
Portnova et al. 2011	Håkon Mosby Mud Volcano, Norwegian Sea	off Norway	1307 m deep						×				
Miljutin et al. 2011	French mining claim, Clarion-Clipperton Fracture Zone, North Pacific Ocean	between Mexico and Hawaii	about 5000 m deep						×				
Liao et al. 2015	Shihlang, Ludao (Green Island), North Pacific Ocean	Taiwan	intertidal sand						×				

**Table 35.** References of *Meyersia major*. Abbreviations: C, citation; D, description; E, ecology; F, feeding and culture; G, genus diagnosis; L, species list of genus; M, morphology; N, DNA taxonomy; O, other experiment; P, population and fauna; R, taxonomic revision; T, taxonomy.

Reference	Collection			T					E		M	O	C
	Locality	Country	Habitat	D	G	R	L	N	P	F			
Hopper 1967	Soldier Key; Pigeon Key, Florida, North Atlantic Ocean	United States	sediment and algae	×									
Hope and Murphy 1972	-	-	-				×						
Gerlach and Riemann 1974	-	-	-				×						
Belogurov and Belogurova 1978a	-	-	-			×							
Belogurov and Belogurova 1978b	-	-	-			×							
Belogurov and Belogurova 1988	-	-	-			×							
Smol and Coomans 2006	-	-	-		×								
Hope 2009	-	-	-						×				
Smol et al. 2014	-	-	-		×								

**Table 36.** References of *Meyersia bandaensis*. Abbreviations: C, citation; D, description; E, ecology; F, feeding and culture; G, genus diagnosis; L, species list of genus; M, morphology; N, DNA taxonomy; O, other experiment; P, population and fauna; R, taxonomic revision; T, taxonomy.

Reference	Collection			T					E		M	O	C
	Locality	Country	Habitat	D	G	R	L	N	P	F			
Kreis 1932 (*A. bandaensis*)	Lontor Island, Banda Sea (in Micoletzky and Kreis 1930)	Indonesia	sand and seagrasses	×									
Kreis 1934(*A. bandaensis*)	same as Kreis 1932			×									
de Coninck 1965(*A. bandaensis*)	-	-	-										×
Hopper 1967	-	-	-			×							
Rachor 1969	-	-	-								×		
Gerlach and Riemann 1974	-	-	-				×						
Belogurov and Belogurova 1978a	-	-	-			×							
Belogurov and Belogurova 1978b	-	-	-			×							
Gourbault and Renaud-Mornant 1990	Fangataufa, Tuamotu Archipelago, South Pacific Ocean	French Polynesia	lagoon sediment						×				

**Table 37.** References of *Meyersia japonica*. Abbreviations: C, citation; D, description; E, ecology; F, feeding and culture; G, genus diagnosis; L, species list of genus; M, morphology; N, DNA taxonomy; O, other experiment; P, population and fauna; R, taxonomic revision; T, taxonomy.

Reference	Collection			T					E		M	O	C
	Locality	Country	Habitat	D	G	R	L	N	P	F			
Yoshimura 1982	Hatake-jima Island, Tanabe Bay, Wakayama, North Pacific Ocean	Japan	intertidal sand	×									
Itani and Shirayama 2001	Tanabe Bay, Wakayama, North Pacific Ocean	Japan	subtidal sand						×				

**Table 38.** References of *Meyersia meridionalis*. Abbreviations: C, citation; D, description; E, ecology; F, feeding and culture; G, genus diagnosis; L, species list of genus; M, morphology; N, DNA taxonomy; O, other experiment; P, population and fauna; R, taxonomic revision; T, taxonomy.

Reference	Collection			T					E		M	O	C
	Locality	Country	Habitat	D	G	R	L	N	P	F			
Kreis 1932 (*A. meridionalis*)	Saparua Island, Banda Sea (in Micoletzky and Kreis 1930)	Indonesia	calcareous algae and lava	×									
Kreis 1934(*A. meridionalis*)	same as Kreis 1932			×									
Schuurmans Stekhoven 1936b(*A. meridionalis*)	-	-	-								×		
Chitwood and Chitwood 1950(*A. meridionalis*)	-	-	-								×		
de Coninck 1965(*A. meridionalis*)	-	-	-										×
Hopper 1967	-	-	-			×							
Rachor 1969	-	-	-								×		
Gerlach and Riemann 1974	-	-	-				×						
Belogurov and Belogurova 1978a	-	-	-			×							
Belogurov and Belogurova 1978b	-	-	-			×							

**Table 39.** References of *Meyersia minor*. Abbreviations: C, citation; D, description; E, ecology; F, feeding and culture; G, genus diagnosis; L, species list of genus; M, morphology; N, DNA taxonomy; O, other experiment; P, population and fauna; R, taxonomic revision; T, taxonomy.

Reference	Collection			T					E		M	O	C
	Locality	Country	Habitat	D	G	R	L	N	P	F			
Hopper 1967	Key Biscayne; Virginia Key, Florida, North Atlantic Ocean	United States	sandy sediment	×									
Hopper and Meyers 1967	Key Biscayne, Florida, North Atlantic Ocean	United States	subtidal sediment						×				
Gerlach and Riemann 1974	-	-	-				×						
Rachor 1969	same as Hopper 1967										×		
Rachor 1970	same as Hopper 1967												×
Maertens and Coomans 1979	-	-	-										×
Chabaud et al. 1983	-	-	-								×		
